# Atomically Dispersed Pt-Ru Dual-Atom Catalysts for Efficient Low-Temperature CO Oxidation Reaction

**DOI:** 10.1007/s40820-025-01997-6

**Published:** 2026-01-05

**Authors:** Yanan Qi, Hongqiu Chen, Feng Hong, Xiangbin Cai, Zhehan Ying, Jiangyong Diao, Zhimin Jia, Jiawei Chen, Ning Wang, Shengling Xiang, Xiaowen Chen, Guodong Wen, Bo Sun, Geng Sun, Hongyang Liu

**Affiliations:** 1 School of Materials Science and Engineering, University of Scienceand Technology of China, Shenyang, 110016 People’s Republic of China; 2 Shenyang National Laboratory for Materials Science, Institute of Metal Research, Chinese Academy of Sciences, Shenyang, 110016 People’s Republic of China; 3 Chongqing Key Laboratory of Chemical Theory and Mechanism, School of Chemistry and Chemical Engineering, Chongqing University, Chongqing, 401331 People’s Republic of China; 4 Division of Physics and Applied Physics, School of Physical and Mathematical Sciences, Nanyang Technological University, Singapore, 637371 Singapore; 5 Materials Characterization and Preparation Facility (GZ), The Hong Kong University of Science and Technology (Guangzhou), Guangzhou, People’s Republic of China; 6 Department of Physics and Center for Quantum Materials, Hong Kong University of Science and Technology, Kowloon, Hong Kong SAR 999077 People’s Republic of China

**Keywords:** CO oxidation, Atomically dispersed, Dual-atom catalysts, Pt-Ru, Synergistic effect

## Abstract

Single-atom catalysts (SACs) have demonstrated excellent performance in heterogeneous catalytic reactions owing to their maximized atomic efficiency, distinctive geometric, and electronic configurations. However, the efficacy of SACs remains limited for certain reactions requiring simultaneous activation of multiple reactants over metallic active sites. Herein, we report an atomically dispersed Pt_1_Ru_1_ dual-atom pair site anchored on nanodiamond@graphene (ND@G) for CO oxidation. The Pt_1_Ru_1_ dual-atom catalyst shows an exceptional turnover frequency (TOF) of 17.6 × 10^−2^ s^−1^ at significantly lower temperature (30 °C), achieving a tenfold increase in TOF compared to single-atom Pt_1_/ND@G catalyst (1.5 × 10^−2^ s^−1^) and surpassing to previously reported Pt-based catalysts under similar conditions. Moreover, the catalyst demonstrates excellent stability, maintaining its activity for 40 h at 80 °C without significant deactivation. The superior catalytic performance of Pt-Ru dual-atom catalysts is attributed to the synergistic effect between Pt and Ru atoms with enhanced metallicity for improving simultaneous adsorption and activation of CO and O_2_, and the tuning of conventional competitive reactant adsorption into a non-competitive pathway over dual-atom pair sites. The present work manifests the advantages of dual-atom pair sites in heterogeneous catalysis and paves the way for precise design of catalysts at the atomic scale.

**Supplementary Information:**

The online version contains supplementary material available at 10.1007/s40820-025-01997-6.

## Highlights

- We successfully fabricated an atomically dispersed dual-atom catalyst featuring Pt_1_-Ru_1_ sites anchored on defective graphene (Pt_1_Ru_1_/ND@G).
- Pt_1_Ru_1_/ND@G achieves a high turnover frequency of 17.6 × 10^−2^ s^−1^ for CO oxidation at 30 °C, which is 10 times higher than Pt_1_/ND@G and demonstrates outstanding performance compared with the previous reports.
- Pt-Ru bond enhances the metallicity of both Pt and Ru atoms, facilitating the simultaneous adsorption and activation of CO and O_2_ and overcoming the limitations of single-atom catalysts.

**Supplementary Information:**

The online version contains supplementary material available at 10.1007/s40820-025-01997-6.

## Graphical abstract

**Figure:**
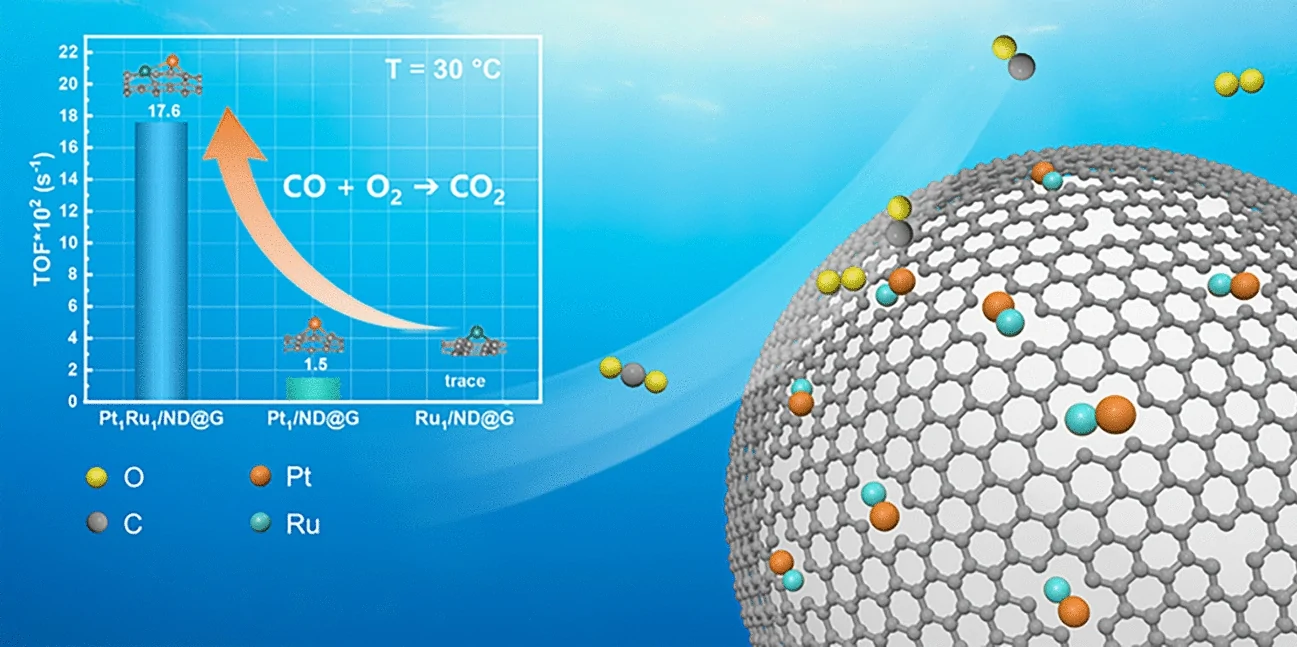

## Introduction

Low-temperature CO oxidation holds significance in the environment and fundamental catalysis due to its crucial application in automobile emission purification [1, 2] and the role as a model reaction in investigating the structure–activity relationship and reaction mechanism [3, 4]. Although precious metal catalysts have been extensively studied for CO oxidation due to their exceptional catalytic activity [5–8], the natural scarcity and high cost impede their practical application. The effective strategy to enhance metal utilization efficiency is to reduce the size of catalyst particle, making single-atom catalysts (SACs) an ideal solution [9–11]. SACs not only achieve near-perfect atom utilization efficiency but also provide highly uniform active sites with distinct geometric coordination and electronic properties. These features enable SACs to outperform conventional nanoparticle catalysts in various reactions, such as selective oxidation and hydrogenation [12–16]. However, it is undeniable that the single-site nature of SACs inherently limits their ability to co-activate multiple reactants simultaneously, such as CO and O_2_ in CO oxidation owing to their competitive adsorption, especially when metal species are supported on inert supports [17, 18]. Additionally, the diminished metallicity in single-atom sites further compromises the catalytic activity [19, 20]. Recent studies have revealed that fully exposed cluster catalysts (FECCs) address the limitations of SACs through spatially adjacent sites, providing diverse structural configurations and versatile catalytic functionalities [21–23]. While this ensemble effect of atomically dispersed sites could enhance the catalytic performance, the variability in sizes and structures of FECCs at the sub-nanometer scale renders the active sites intricate, which complicates the investigation of the structure–activity relationship and underlying reaction mechanism.

Therefore, dual-atom catalysts featuring active sites composed of two metal atoms have garnered significant attention [24, 25]. These catalysts can effectively integrate the advantages of the aforementioned two types of catalysts. The adjacent metal atoms modulate the electronic structure and optimize the physicochemical properties of the metal species, providing more possibilities for the activation and adsorption of the reactants [26–30]. On this basis, heteronuclear dual-atom catalysts are expected to become an attractive alternative in heterogeneous catalysis, which may bring about new catalytic functions through the synergistic effect between two metal atoms with different properties. Currently, reported synthesis strategies for constructing dual-atom catalysts include “precursor-preselected” wet-chemistry approaches [31], “metal ion recognition” [32], “anion replacement deposition–precipitation” [33], etc. However, these methods typically rely on expensive ligands and involve complex procedures, which significantly limit the scalability and practical application of dual-atom catalysts.

Herein, we developed an effective strategy to construct Pt_1_Ru_1_ dual-atom pair sites anchored on defect-rich graphene support via a co-impregnation method. Ruthenium (Ru) was selected as the auxiliary atom in the designed bimetallic catalyst due to its extensive application in oxidation reactions [34, 35] and its unique synergy with platinum. Among the various transition metals explored as promoters for Pt-based catalysts, Ru exhibits distinctive advantages in CO oxidation. Although Fe, Co, and Ni can also influence the electronic structure of Pt, their promotional effects are generally less stable under oxidative conditions. In contrast, Ru interacts strongly with Pt, leading to an optimized d-band center and enhanced charge redistribution, which not only adjusts CO adsorption but also facilitates O_2_ activation [36, 37]. In our previous work, Pt_n_/ND@G exhibited exceptional activity for low-temperature CO oxidation [23]. However, when the particle size was reduced to a single atom, a significant decrease in catalytic activity was observed. To overcome this limitation, we rationally designed Ru-incorporated single-atom Pt catalysts to construct Pt-Ru dual-atom pair site (DAPS). The obtained Pt_1_Ru_1_/ND@G catalyst exhibits remarkable enhancement in CO oxidation activity compared to its single-atom counterparts. At 30 °C, the turnover frequency (TOF) of Pt_1_Ru_1_/ND@G reaches 17.6 × 10^−2^ s^−1^, representing a tenfold improvement over Pt_1_/ND@G (1.5 × 10^−2^ s^−1^) and exceeding the performance of previously reported Pt-based catalysts under similar conditions. This exceptional low-temperature activity originates from the synergistic effects between Pt and Ru in the DAPS. 

The structural characterization of Pt_1_Ru_1_/ND@G was systematically investigated by high-angle annular dark-field scanning transmission electron microscopy (HAADF-STEM) and extended X-ray absorption fine structure (EXAFS) spectra, which confirmed the synthesis of Pt_1_Ru_1_ DAPS and the formation of Pt-Ru bonds. Notably, the electronic interaction between Pt and Ru increased their respective metallicity, as verified by X-ray photoelectron spectroscopy (XPS) and X-ray absorption near-edge structure (XANES) results. Comprehensive characterizations combined with theoretical calculations demonstrated that the excellent CO oxidation performance of Pt_1_Ru_1_/ND@G is attributed to the simultaneous enhancement of CO and O_2_ adsorption and activation, resulting from the unique geometric construction and electronic state of Pt_1_Ru_1_ DAPS. This work paves the way for the application of atomically dispersed hetero dual-atom pair sites in catalysis.

## Experimental Section

### Materials

The ND@G support was fabricated using nanodiamond (ND) powders purchased from Beijing Grish Hitech Co. (China). Analytical-grade chloroplatinic acid hexahydrate (H_2_PtCl_6_·6H_2_O), palladium nitrate dihydrate (Pd(NO_3_)_2_·2H_2_O), and ruthenium chloride trihydrate (RuCl_3_·3H_2_O) were obtained from Aladdin Chemical Reagent Inc. Copper nitrate trihydrate (Cu(NO_3_)_2_·3H_2_O), ferric nitrate nonahydrate (Fe(NO_3_)_3_·9H_2_O), and cobalt nitrate hexahydrate (Co(NO_3_)_2_·6H_2_O) were purchased from Sinopharm Co., Ltd. ND@G was prepared by annealing commercial ND powders in a furnace under an argon flow (100 mL/min) at 1100 °C for 4 h. The obtained black ND@G powders were further purified by concentrated hydrochloric acid and thoroughly washed with deionized water.

### Preparation of Pt_1_Ru_1_/ND@G, Pt_1_/ND@G, Ru_1_/ND@G, and PtM/ND@G Catalysts

#### ***Preparations of the Pt***_***1***_***Ru***_***1***_***/ND@G***

Pt_1_Ru_1_/ND@G was prepared by the co-impregnation strategy. The nominal contents of Pt and Ru were 0.1 and 0.05%, respectively. A certain amount of H_2_PtCl_6_·6H_2_O solution and RuCl_3_·3H_2_O solution was added to 3 mL of anhydrous ethanol. Then, 200 mg of ND@G powders was dispersed into the solution and stirred well until the ethanol was completely evaporated. The samples were obtained after drying in a vacuum at 60 °C for 12 h. The dried samples were pretreated at 300 °C for 1 h in a mixed gas atmosphere (48% H_2_, 1% CO, 1% O_2_, balanced with He).

The synthesis method for other Pt-Ru catalysts with different nominal contents is the same as described above, merely by altering the amount of Ru added.

#### ***Preparations of Pt***_***1***_***/ND@G and Ru***_***1***_***/ND@G***

Pt_1_/ND@G and Ru_1_/ND@G were synthesized by an impregnation method. The detailed procedure is similar to that for Pt_1_Ru_1_/ND@G. 0.15Pt/ND@G and 0.15Ru/ND@G can be prepared by changing the amount of the metal solution.

#### Synthesis of PtM/ND@G Catalysts

The bimetallic catalysts PtPd/ND@G, PtFe/ND@G, PtCo/ND@G, and PtCu/ND@G were synthesized via co-impregnation with a controlled Pt/M molar ratio of 1:1. The detailed procedure is similar to that for Pt_1_Ru_1_/ND@G.

### Catalyst Characterization Methods

X-ray diffraction (XRD) patterns were collected using an X-ray diffractometer (Bruker D8 Advance) with a Cu target at a scan rate of 2° min^−1^ to characterize the crystal structures of the samples. HAADF-STEM images were recorded by a JEOL JEM ARM 200CF aberration-corrected scanning transmission electron microscope at an accelerating voltage of 200 kV. X-ray photoelectron spectroscopy (XPS) analysis was carried out with an ESCALAB 250 instrument with Al Kα X-rays (1486.6 eV, 150 W), using a pass energy of 50.0 eV for high-resolution spectra. Metal loadings of Pt and Ru in catalysts were detected by inductively coupled plasma atomic emission spectrometer (ICP-OES: Agilent 5110). X-ray absorption spectroscopy (XAS) spectra were recorded on beamline 1W1B of the Beijing Synchrotron Radiation Facility, which operated at 2.5 GeV with a current of 250 mA. A Si (111) double-crystal monochromator was used for measurements. Data were collected in transmission mode using ionization chambers as detectors. The spectra were acquired under ambient conditions. All XAS data processing and analysis were processed and analyzed using the Demeter software package [38].

In situ CO adsorption diffuse reflectance infrared Fourier-transform infrared spectroscopy (DRIFTS) experiments were performed on a Thermo Scientific Nicolet IS10 Fourier-transform infrared spectrometer equipped with an MCT detector cooled with liquid nitrogen. The resolution of the spectrometer was 4 cm^−1^ over 64 scans. The corresponding spectra were recorded over 25 min during He purging after CO saturation adsorption for 30 min.

Temperature-programmed desorption of CO (CO-TPD) was carried out under a He atmosphere. The samples were pretreated at 300 °C for 1 h under a mixed gas atmosphere (48% H_2_, 1% CO, 1% O_2_, balanced with He), and then, the adsorption of CO was performed at room temperature for 1 h in 1% CO/99% He (15 mL min^−1^). The samples were heated at a rate of 2 °C min^−1^ after purging with He (15 mL min^−1^) for 1 h.

CO–O_2_ transient response (CO–O_2_–TR) experiments with Pt_1_/ND@G, Ru_1_/ND@G, and Pt_1_Ru_1_/ND@G catalysts were performed to investigate the CO and O_2_ adsorption capacities of different samples. First, the catalysts were exposed to CO until saturation in 5% CO/95% He (15 mL min^−1^) at room temperature, followed by purging with He (15 mL min^−1^) to remove any physically adsorbed CO. After the CO adsorption pretreatment, the system was switched to 1% O_2_/99% He (15 mL min^−1^) at room temperature for 1 h (with MS signals of *m*/*z* 28 and 44 being recorded).

### Reaction Evaluation

CO oxidation was performed at ambient pressure in a temperature-programmed mode with a heating rate of 1 K min^−1^ over a temperature range from 30 to 300 °C. The reaction gas feed consisted of 1% CO, 1% O_2_, and 98% He. Before the reaction, the catalyst (20–50 mg) was diluted with 250 mg quartz and reduced in a mixed gas atmosphere (48% H_2_, 1% CO, 1% O_2_, balanced with He) at 300 °C for 1 h. The reactants and products were analyzed by an online gas chromatograph (Agilent 7890B). The space velocity is 48,000 mL g_cat_^−1^ h^−1^ for the catalytic performance test and 18,000 mL g_cat_^−1^ h^−1^ for the stability test. The TOF value was separately measured by keeping the substrate conversion below 15%, and the calculation of the TOF was based on the real Pt loading by an ICP analysis in the catalyst. During the kinetic measurements, the reactor was heated to 30 °C in a gas mixture of 5% CO, pure O_2_, and He. To investigate the effect of CO and O_2_ partial pressure on the reaction rate, the partial pressure was varied between 1 and 4 kPa. Each cycling test was terminated at 200 °C and subsequently cooled to ambient temperature.

### Details of Theoretical Calculations

The density functional theory (DFT) calculations are carried out using the VASP (Vienna Ab initio Simulation Package) package to understand the reaction mechanisms and energetics [39]. The energy cutoff for plane wave basis sets is 400 eV [40], and PAW pseudopotentials were used for describing the effective potential of nuclei and core electrons [41]. Spin-polarized calculations are always applied throughout this manuscript. PBE functional is exploited for the exchange–correlation functional of electrons [42], and the Grimme DFT-D3 method implemented in VASP is also employed to account for the vdW interactions [43]. The structure optimization is performed until the forces on all atoms are less than 0.03 eV Å^−1^. The transition states are obtained using the CI-NEB method and DIMER method [44–46]. Thermodynamic corrections are applied at conditions of 323.15 K and specified pressures to more accurately reflect the effects of temperature and pressure: The thermodynamic correction for CO at *T* = 323.15 K, *P* = 1.0 × 10^3^ Pa was calculated as − 0.78 eV; for O₂ at *T* = 323.15 K, *P* = 2.0 × 10^3^ Pa, it was − 0.78 eV; and for CO_2_ at *T* = 323.15 K, *P* = 1.0 × 10^2^ Pa, it was − 0.91 eV.

The adsorption energy is defined as follows:

1 $$E_{{{\mathrm{ads}}}} = E_{{{\mathrm{total}}}} - E_{{{\mathrm{slab}}}} - E_{{{\mathrm{adsorbent}}}}$$

where *E*_total_ is the total energy of the adsorbate adsorbed on the substrate material, *E*_slab_ is the energy of the substrate material, and *E*_ads_ is the energy of the adsorbate.

The Gibbs free energy is defined as follows:

2 $$G\left( {T,P} \right) = E^{{{\mathrm{DFT}}}} + \Delta G_{{{\mathrm{corr}}}}$$

where *E*^DFT^ is the energy calculated by DFT at 0 K, and Δ*G*_corr​_ is the thermodynamic correction term, which accounts for the effects of temperature and pressure on the adsorption energy, thereby enabling accurate prediction of the chemical reaction free energy under actual reaction conditions.

## Results and Discussion

### Structural Characterizations

**Fig. 1:**
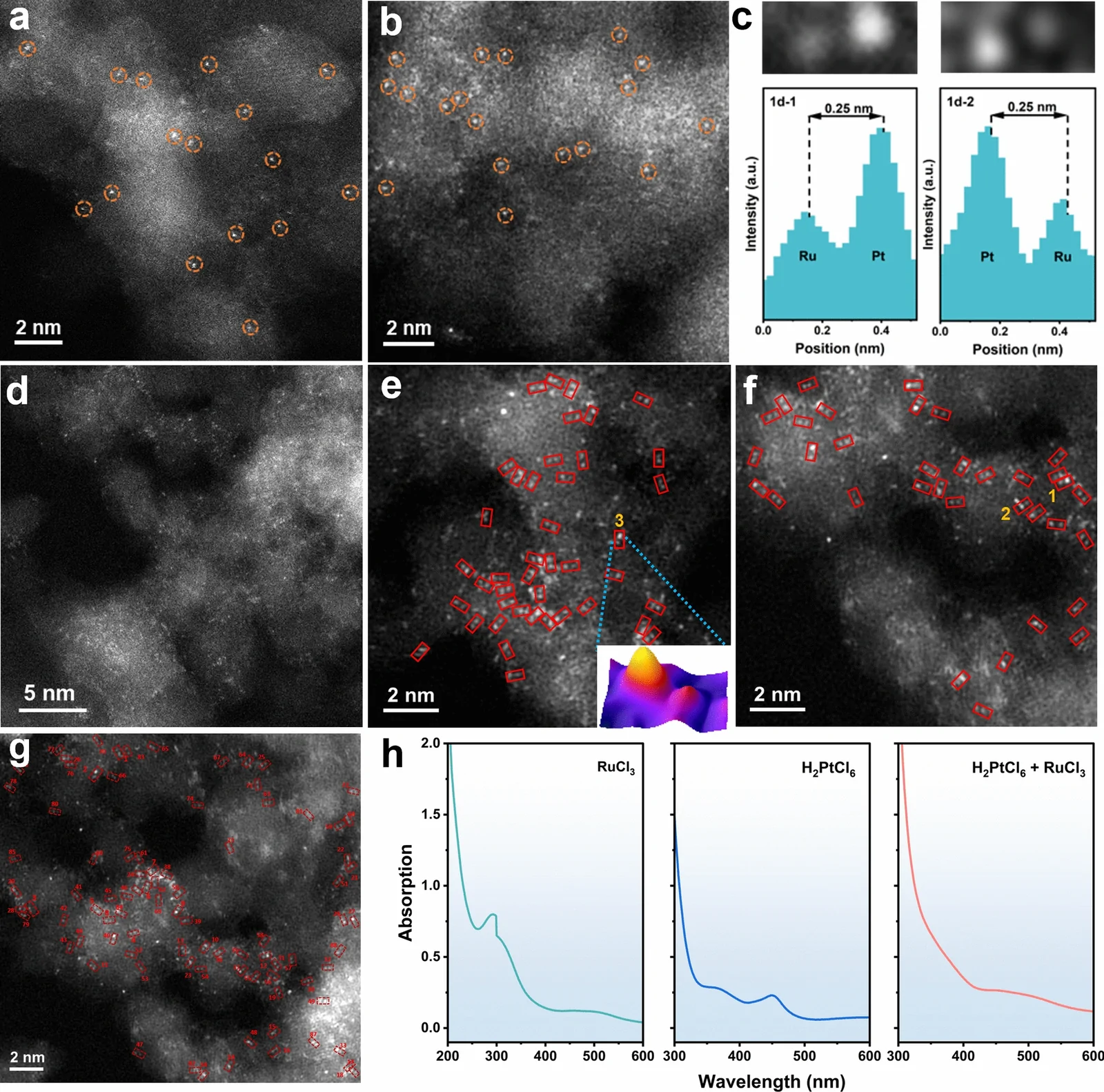
HAADF-STEM images of **a** Pt_1_/ND@G, **b** Ru_1_/ND@G. **c** Enlarged images and corresponding intensity profiles of rectangles 1 and 2 in image **f**. **d–f** HAADF-STEM images of Pt_1_Ru_1_/ND@G (inset in **e**: 3D intensity surface plot of rectangle 3). **g** Statistics of atomic pairs in the HAADF-STEM images of Pt-Ru diatomic samples.** h** UV–Vis spectra of the precursor solutions (The sudden change at the 300 nm position for the UV–Vis spectrum of RuCl_3_ is due to a switching of the light source)

The Pt_1_/ND@G, Ru_1_/ND@G, and Pt_1_Ru_1_/ND@G catalysts were prepared using an impregnation method, with H_2_PtCl_6_ and RuCl_3_ as precursors. In aqueous solution, the PtCl₆^2−^ anion and Ru^3+^ cation interact electrostatically, facilitating the formation of Pt-Ru dual-atom pair sites on the defective ND@G surface [25, 47, 48]. The UV–Vis spectrum (Fig. 1h) indicates the presence of inter-ionic interactions among the precursors in the solution. Subsequently, the excess Cl^−^ ions were removed through reductive treatment. Detailed procedures were described in the Methods section. The actual Pt and Ru loadings in the Pt_1_/ND@G, Ru_1_/ND@G, and Pt_1_Ru_1_/ND@G samples were determined by inductively coupled plasma optical emission spectrometry (ICP-OES), which were basically consistent with the nominal content (Table S1). The X-ray diffraction (XRD) patterns of the above three samples showed no detectable diffraction peaks for Pt or Ru due to their low loadings and high dispersion over the support surface (Fig. S1). The Pt_1_Ru_1_ catalyst was collected after a CO oxidation reaction and subsequently characterized by XRD to assess its structural integrity. No distinct diffraction peaks attributable to metal species were detected.

In addition, the aberration-corrected HAADF-STEM was employed to elucidate the atomic-scale structure of these catalysts. The HAADF-STEM images of Pt_1_/ND@G and Ru_1_/ND@G confirmed the single-atom dispersion of Pt and Ru atoms (Figs. 1a, b, and S2-S3). Strikingly, the Pt_1_Ru_1_/ND@G sample exhibited numerous spatially adjacent Pt-Ru DAPS, as highlighted in the red rectangular boxes (Fig. 1e, f). Due to the difference in *Z*-contrast, Pt atoms appeared marginally brighter than the Ru atoms. We conducted a qualitative analysis of the proportion of Pt-Ru atomic pairs in the sample (as shown in Fig. 1g) and found that over 70% of the observed metal species formed atomic pairs. The average distance between Pt and Ru in Pt_1_Ru_1_/ND@G was approximately 2.53 Å in the intensity profiles by counting 50 dual-atom pair sites (Fig. S4). This distance is slightly shorter than the sum of the atomic radii of Pt and Ru, indicating the formation of a Pt-Ru bond.

To further verify the formation of Pt-Ru bond and electronic interaction between Pt and Ru, X-ray photoelectron spectroscopy (XPS) spectra of Pt 4*f* and Ru 3*p* were recorded. The binding energy shifts reflect the perturbation of the electronic states of the atoms. A doublet band centered at 71.7 and 75.0 eV was observed in the Pt_1_/ND@G sample due to the spin–orbit splitting (Fig. S5). A shift of 0.3 eV toward lower binding energy was observed for Pt 4*f* peaks in Pt_1_Ru_1_/ND@G, indicating an increased metallicity of Pt atoms. Similarly, the same trend was observed for the Ru 3*p* peaks in the Ru_1_/ND@G and Pt_1_Ru_1_/ND@G samples (Fig. S6). These results confirmed that the electronic interaction between Pt and Ru atoms in Pt_1_Ru_1_ DAPS increased both electronic state densities through the Pt–Ru bond.

**Fig. 2:**
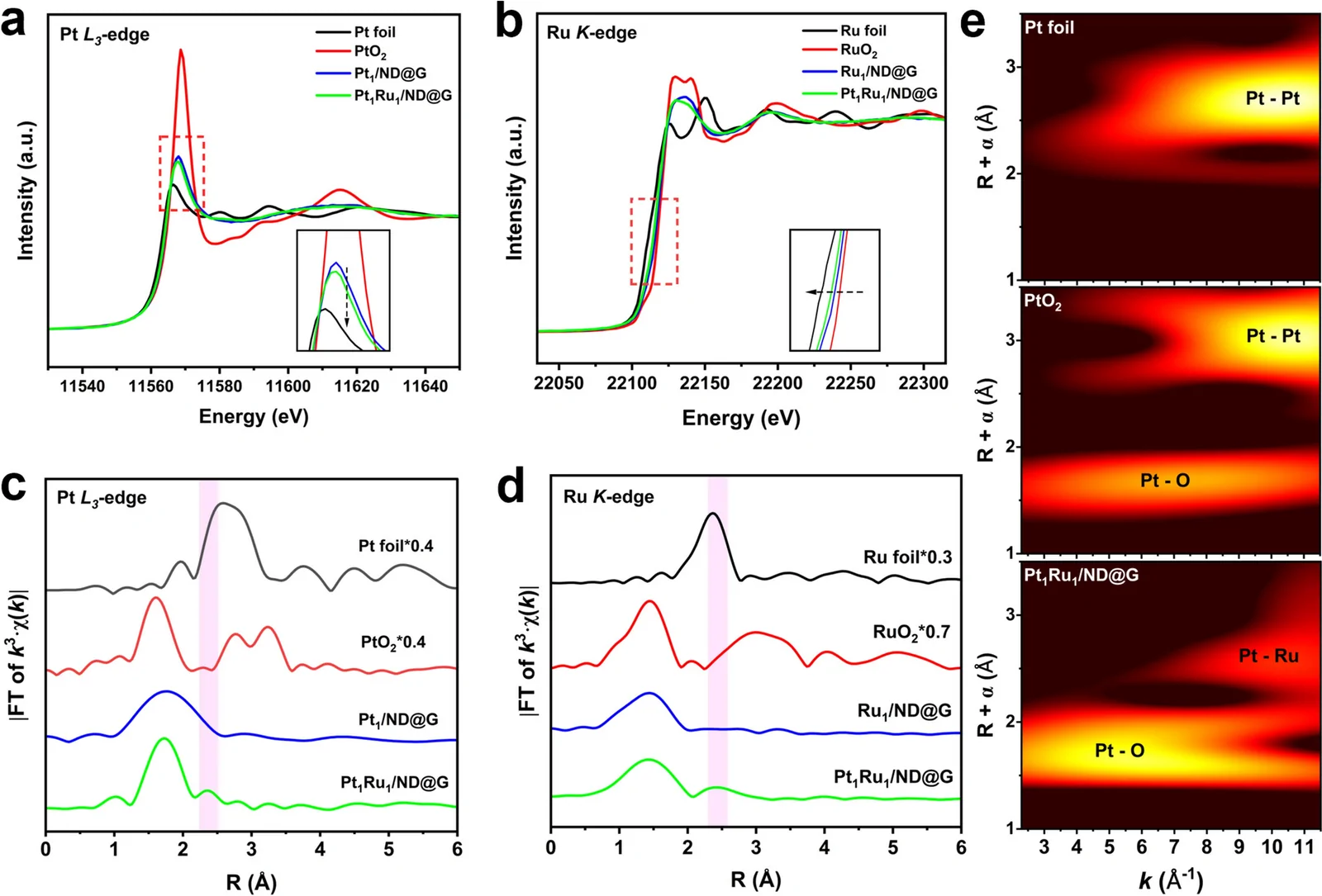
**a** Normalized Pt L-edge XANES spectra and **c** corresponding FT-EXAFS spectra (without phase correction) for Pt₁/ND@G and Pt_1_Ru_1_/ND@G. **b** Normalized Ru* K*-edge XANES spectra and **d** corresponding FT-EXAFS spectra (without phase correction) for Ru₁/ND@G and Pt_1_Ru_1_/ND@G. **e** Wavelet transform (WT) analysis of Pt_1_Ru_1_/ND@G and reference samples

Moreover, X-ray absorption spectroscopy (XAS) was further employed to investigate the electronic structure and coordination environment of Pt and Ru atoms in Pt_1_/ND@G, Ru_1_/ND@G, and Pt_1_Ru_1_/ND@G catalysts. Figure 2a shows the normalized X-ray absorption near-edge structure (XANES) spectra at the Pt *L*_3_*-*edge of these samples, where the white line intensities for Pt_1_/ND@G and Pt_1_Ru_1_/ND@G were situated between those of Pt foil and PtO_2_, indicating that the Pt species in both samples are positively charged. However, the reduced white line intensity observed for Pt_1_Ru_1_/ND@G compared to that of Pt_1_/ND@G indicates a lower oxidation state of Pt atoms within Pt-Ru DAPS. Analogously, the Ru *K*-edge XANES spectra (Fig. 2b) revealed absorption thresholds in Ru_1_/ND@G and Pt_1_Ru_1_/ND@G located in the range of Ru foil and RuO_2_, implying the electron-deficient state of Ru species. The Ru *K*-edge absorption threshold in Pt_1_Ru_1_/ND@G was lower than that in Ru_1_/ND@G, demonstrating a decrease in the Ru oxidation state of Pt-Ru DAPS. The XANES results of mutually promoted metallicity in Pt_1_Ru_1_ DAPS are consistent with the XPS results. Increased electronic state density of Pt_1_Ru_1_ DAPS can not only enhance the activation of CO due to the increased *d*–*π* backbonding between metal sites and CO [20, 49], but also improve the O_2_ activation because of the facile formation of negatively charged active oxygen species at dual-atom pair sites with sufficient electron density [50, 51].

The geometric construction of Pt_1_Ru_1_/ND@G was further investigated by extended X-ray absorption fine structure (EXAFS) spectra. The local structure information of Pt and Ru atoms in Pt_1_/ND@G, Ru_1_/ND@G, and Pt_1_Ru_1_/ND@G was extracted from their Fourier transforms of EXAFS (FT-EXAFS) data (Fig. 2c, d). Detailed fitting parameters are provided in Table S4. For Ru_1_/ND@G, the coordination number (C.N.) of Ru–C/O (metallic) at ∼ 2.02 Å was 4.5, with no detectable Ru–Ru scattering path, confirming the single-atom dispersion of Ru. Similarly, Pt_1_/ND@G also exhibited no observable Pt–Pt bonding. In addition to the dominant Pt-C/O and Ru–C/O contributions, Pt_1_Ru_1_/ND@G showed distinct shoulder peaks at both the Pt *L*_3_-edge (2.52 Å) and Ru *K*-edge (2.58 Å), providing direct evidence for the formation of Pt-Ru bond. The wavelet transform (WT) analysis of Pt *L*-edge EXAFS oscillations further confirmed the coordination structure of the Pt-Ru DAPS (Fig. 2e). The EXAFS fitting curves for the samples are displayed in Figs. S12–S14. The fitting results reveal a Ru-Pt coordination number of 0.9 with an average bond distance of 2.58 Å in Pt_1_Ru_1_/ND@G sample, manifesting the dominance of Pt-Ru DAPS. Moreover, the Pt_1_Ru_1_/ND@G catalyst exhibited reduced coordination numbers for M-C/O bonds compared to Pt_1_/ND@G and Ru_1_/ND@G, resulting in enhanced metallicity of the Pt and Ru species.

### Catalytic Performance for CO Oxidation

**Fig. 3:**
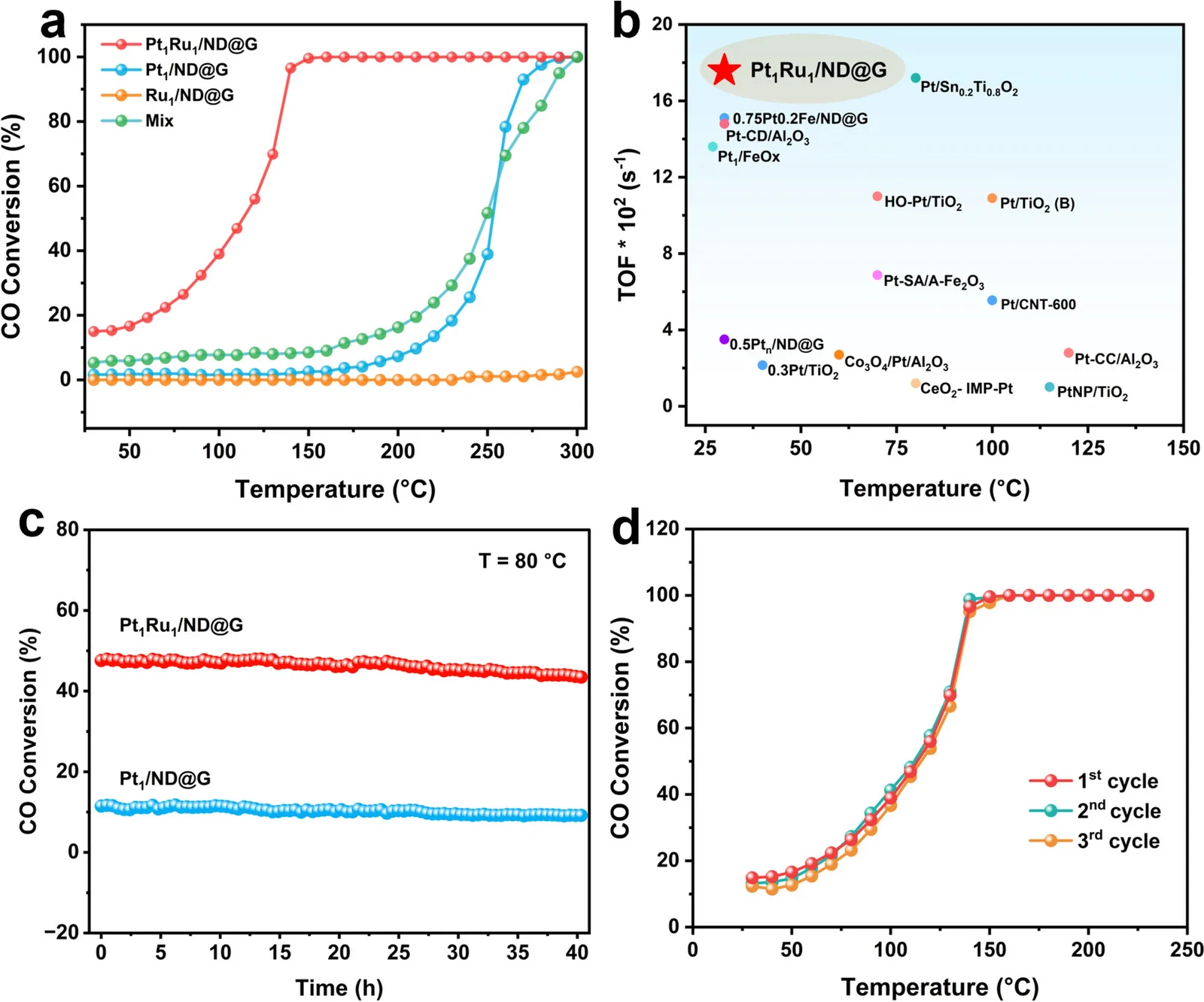
**a** Catalytic performance (1 vol% CO, 1 vol% O_2_, balanced with He; GHSV = 48,000 mL g_cat_^−1^ h^−1^) of Pt_1_/ND@G, Ru_1_/ND@G, Pt_1_Ru_1_/ND@G, and the physically mixed catalyst. **b** Comparison of reported TOFs over various catalysts under similar reaction conditions. **c** Stability test of Pt_1_Ru_1_/ND@G and Pt_1_/ND@G at 80 ℃ (GHSV = 18,000 mL g_cat_^−1^ h^−1^). **d** Cycling test of the CO oxidation activity for Pt_1_Ru_1_/ND@G

The catalytic activities of as-synthesized single-atom site (Pt_1_, Ru_1_) and Pt_1_Ru_1_ dual-atom pair site catalysts were systematically evaluated for the CO oxidation reaction to investigate their structure–activity relationship. In Fig. 3a, CO conversion profiles are shown as a function of reaction temperature. At a gas hourly space velocity of 48,000 mL g_cat_^−1^ h^−1^, the Pt_1_Ru_1_/ND@G catalyst exhibited the highest CO conversion at 30 °C and the lowest temperature for complete conversion (160 °C) compared with Pt_1_/ND@G and Ru_1_/ND@G. In contrast, complete CO conversion required a temperature up to 300 °C on Pt_1_/ND@G, which is much higher than that of Pt_1_Ru_1_/ND@G, reflecting the superior CO oxidation performance of the Pt_1_Ru_1_ DAPS. Furthermore, Ru_1_/ND@G exhibited almost no activity below 300 °C, indicating that isolated Ru species are inactive for CO oxidation. A series of Pt-Ru bimetallic catalysts with different Ru contents were prepared and evaluated for CO oxidation (Fig. S7). The CO oxidation activity (T_50_ or T_100_) exhibited an inverse volcano-type dependence on the Ru content (Fig. S8). It can be observed that a further increase in Ru content leads to a decline in catalytic activity. This might be attributed to the excessive addition of Ru causing aggregation among Ru atoms [52], resulting in a reduction of Pt-Ru dual-atom pair sites and a deterioration of performance. To investigate the influence of different metal atoms on Pt single-atom catalysts, we synthesized a series of bimetallic catalysts, including PtPd/ND@G, PtFe/ND@G, PtCo/ND@G, and PtCu/ND@G catalysts with a Pt/M molar ratio of 1:1. The CO oxidation performance was evaluated under identical conditions (Fig. S9). Notably, Ru-modified Pt_1_/ND@G exhibited significantly enhanced activity compared to catalysts incorporating other metal atoms (e.g., Pd, Fe, Co, Cu).

To verify the critical role of the synergistic effect of Pt-Ru DAPS in CO oxidation, the physically mixed sample (Pt_1_/ND@G + Ru_1_/ND@G) was evaluated in the CO oxidation reaction (In the physically mixed samples, the Pt_1_ and Ru_1_ sites are theoretically not adjacent). It was found that, under the same experimental conditions, the catalytic performance of the physically mixed sample (Pt_1_/ND@G + Ru_1_/ND@G) was similar to that of Pt_1_/ND@G, significantly lower than that of Pt_1_Ru_1_/ND@G, highlighting the importance of adjacent Pt-Ru DAPS in CO oxidation reaction (Fig. 3a). Although both Pt_1_/ND@G and Pt_1_Ru_1_/ND@G exhibit residual Pt–Cl coordination in XAFS, their coordination numbers are comparable, yet they exhibit significant differences in catalytic performance. Therefore, we attribute the enhanced activity to the crucial role of the Pt–Ru bond. To further quantify the activity of Pt_1_Ru_1_ catalyst, the turnover frequency of CO oxidation over the Pt sites was measured and compared to the results reported in literatures (Fig. 3b and Table S5). Under similar conditions, our work exhibits higher activity compared to previously reported efficient Pt-based catalysts for CO oxidation. Previous studies have found that changes in site density can affect the performance of catalysts. Therefore, to further illustrate the enhancing effect of Ru atoms on the performance of Pt_1_/ND@G, we synthesized single-metal catalysts with the same total metal loading as Pt_1_Ru_1_/ND@G and compared their catalytic activities (Fig. S10). The results indicated that the TOF of 0.15 Pt/ND@G at 30 °C was significantly lower than that of Pt_1_Ru_1_/ND@G, demonstrating that the observed performance cannot be attributed merely to variations in site density.

The stability tests of the Pt_1_Ru_1_ catalyst and Pt single-atom catalyst were carried out at 80 °C, and it was observed that the catalyst maintained its activity without obvious deactivation over an extended period, demonstrating excellent stability (Fig. 3c). The samples after the stability test were collected and characterized by HAADF-STEM. No significant agglomeration was observed, and a large number of atomic pair structures were still present (Fig. S18). Moreover, Pt_1_Ru_1_ catalyst showed no significant decline in activity after three cycles (Fig. 3d). Finally, we performed a comparative analysis between Pt_1_Ru_1_/ND@G and Pt-Ru catalysts supported on commercial carriers (Fig. S11) and observed that Pt_1_Ru_1_/ND@G exhibited an obvious performance improvement in the low-temperature range.

### CO Oxidation Mechanism Studies

**Fig. 4:**
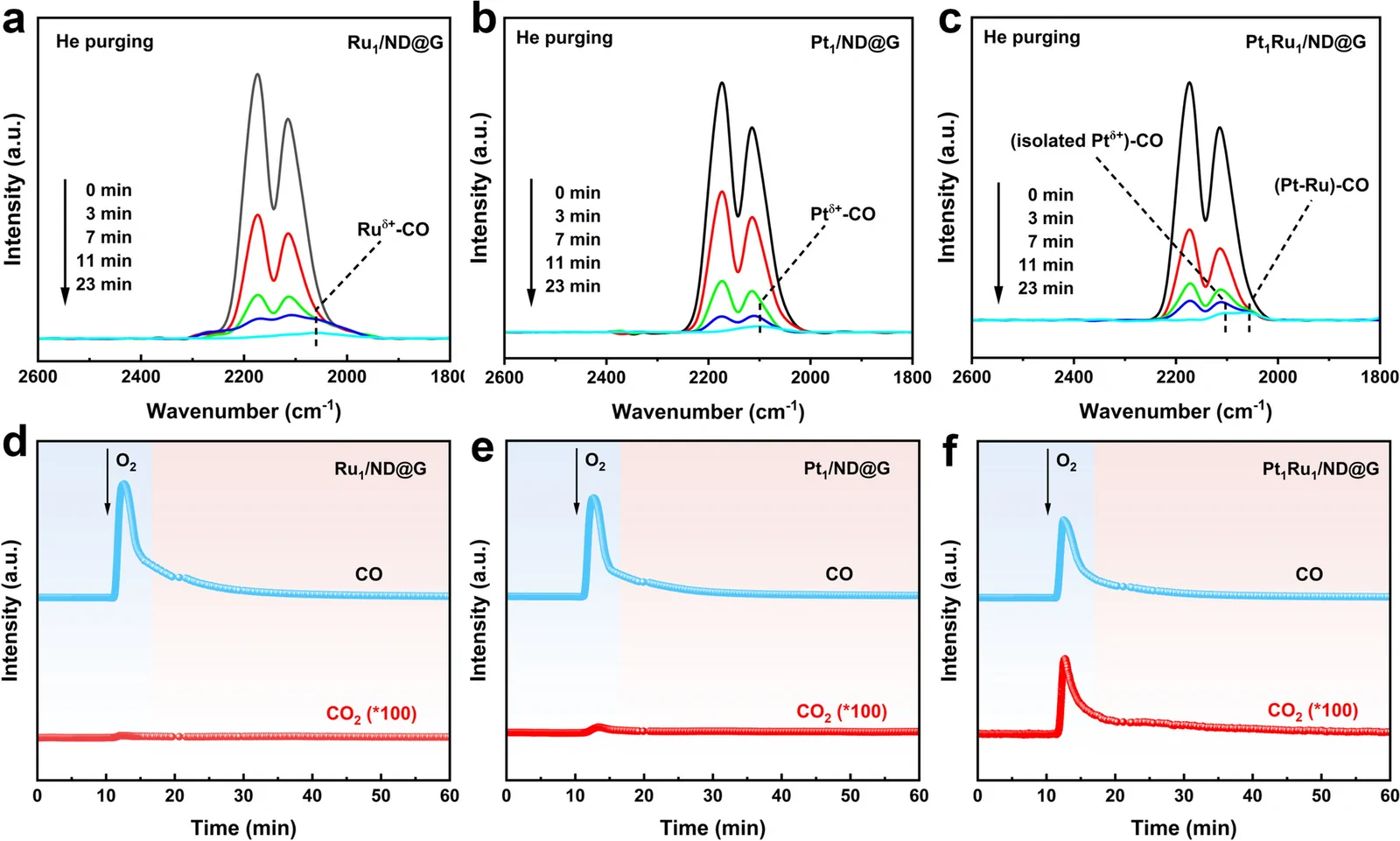
**a–c** In situ DRIFTS of CO adsorption, **d–f** CO–O_2_ transient response (CO–O_2_–TR) experiments of Ru_1_/ND@G, Pt_1_/ND@G, and Pt_1_Ru_1_/ND@G

The in situ diffuse reflectance infrared Fourier-transform (DRIFT) spectroscopy of CO adsorption was carried out to further determine the geometric construction and electronic structure of these catalysts due to the sensitivity of CO adsorption to the geometric and electronic properties of active sites. All samples were pre-exposed to the CO flow at 30 °C for 30 min, followed by purging with a He flow. As shown in Fig. 4a–c, the bands at 2175 and 2115 cm^−1^ were related to the gaseous CO [53, 54] and gradually diminished with increasing He purge time. In addition, the peaks located at 2060 cm^−1^ for Ru_1_/ND@G (Fig. 4a) and 2098 cm^−1^ for Pt_1_/ND@G (Fig. 4b) were attributed to the linear adsorption of CO on positively charged Pt and Ru atoms, respectively [22, 23, 55]. Due to the weak adsorption strength of CO on single atom and the low metal loading, the CO adsorption bands on both Pt_1_/ND@G and Ru_1_/ND@G catalysts were weak. Compared with single-atom Pt_1_ and Ru_1_ samples, two peaks centered at 2056 and 2098 cm^−1^ were observed on Pt_1_Ru_1_/ND@G sample (Fig. 4c). Combined with the results of DFT calculations (Fig. 6d), the two peaks can be assigned to the linear adsorption of CO on Pt species of Pt_1_Ru_1_ pairs and a small number of isolated Pt species. This observation indicates enhanced metallicity of the Pt species in Pt_1_Ru_1_ DAPS, which is consistent with the results of XANES and XPS.

**Fig. 5:**
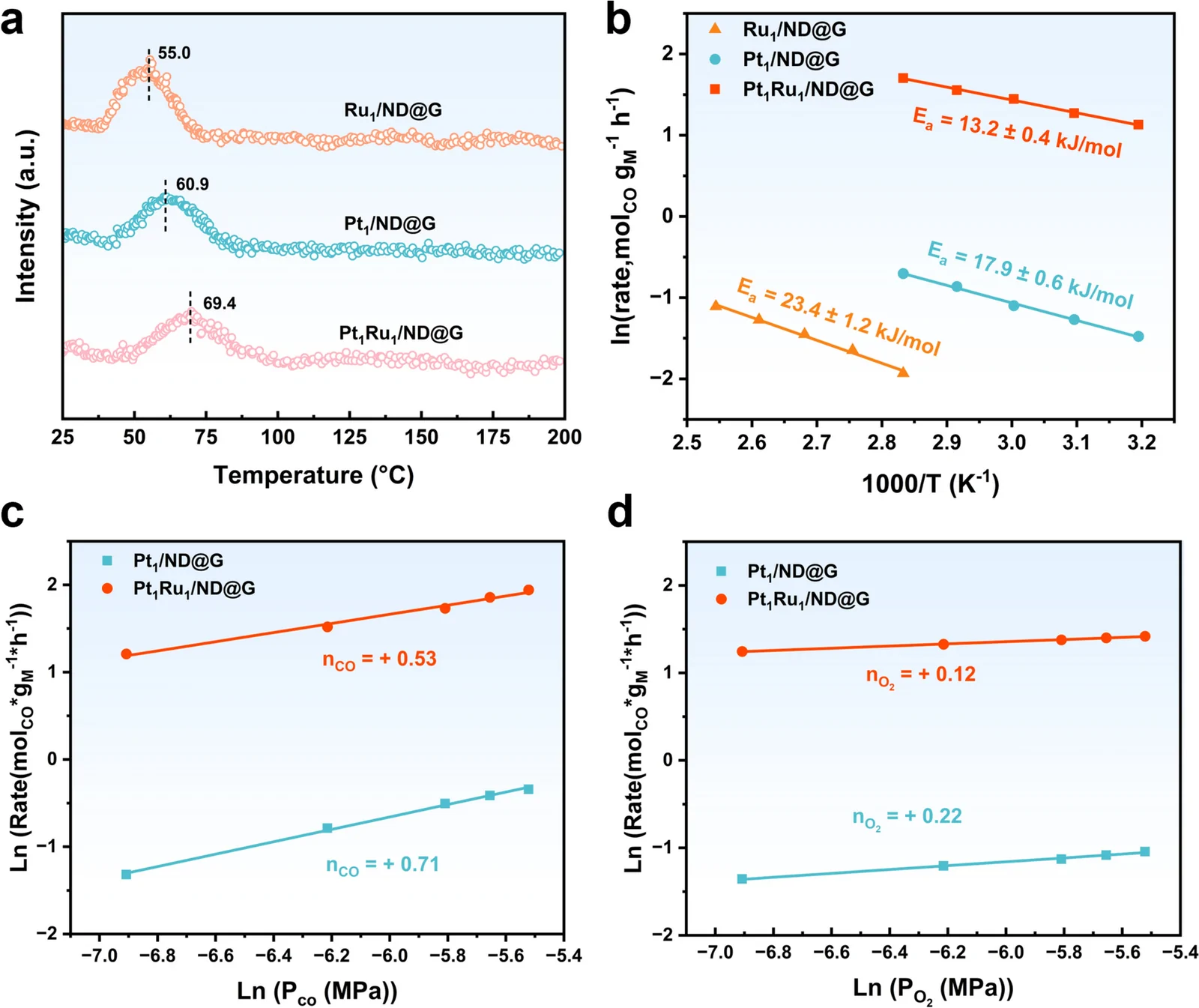
**a** CO-TPD spectra and **b** apparent activation energies of Ru_1_/ND@G, Pt_1_/ND@G, and Pt_1_Ru_1_/ND@G. Reaction orders for **c** CO and **d** O_2_ of Pt_1_/ND@G and Pt_1_Ru_1_/ND@G

To further investigate the adsorption behavior of two reactants (CO and O_2_) and the reaction mechanism on the surface of the catalysts, we conducted CO-O_2_ transient response experiments (Fig. 4d–f). The samples were first pretreated with 5% CO/He until reaching adsorption saturation, followed by He purging to remove the physically adsorbed CO species. When the gas was switched to 1% O_2_/He, a distinct peak appeared on the CO concentration curve (with an MS signal of *m/z* = 28) for all samples, resulting from the competitive adsorption between CO and O_2_. The CO peak intensity of Pt_1_Ru_1_/ND@G is lower than that of Pt_1_/ND@G and Ru_1_/ND@G, indicating that the Pt-Ru DAC has a stronger CO adsorption. Upon the introduction of O_2_, we also found that a distinct CO_2_ signal emerged on the concentration curve (with an MS signal of *m/z* = 44) of Pt_1_Ru_1_/ND@G. By contrast, only a weak CO_2_ peak was observed for Pt_1_/ND@G, and virtually none for Ru_1_/ND@G. The production trend of CO_2_ corresponds well with the catalytic performance as discussed in Fig. 3a. Furthermore, the CO-TPD profiles of Pt_1_/ND@G, Ru_1_/ND@G, and Pt_1_Ru_1_/ND@G were recorded to investigate the strength of CO adsorption on these samples (Fig. 5a). The higher CO desorption temperature on Pt_1_Ru_1_/ND@G than Pt_1_/ND@G and Ru_1_/ND@G indicates a stronger CO adsorption strength over Pt_1_Ru_1_ DAC, contributing to a superior CO oxidation activity.

Kinetic measurements were conducted to explore the reaction mechanism of CO oxidation on Pt_1_Ru_1_/ND@G. The apparent activation energies derived from Arrhenius plots (Fig. 5b) reveal that Pt_1_Ru_1_/ND@G exhibits a lower activation barrier (13.2 kJ mol^−1^) compared to Pt_1_/ND@G (17.9 kJ mol^−1^) and Ru_1_/ND@G (23.4 kJ mol^−1^), which is consistent with its superior low-temperature activity. Additionally, Fig. 5c, d shows the reaction orders of CO and O_2_ for Pt_1_/ND@G and Pt_1_Ru_1_/ND@G samples. Compared with Pt_1_/ND@G, Pt_1_Ru_1_/ND@G has a lower reaction order of CO (0.53 versus 0.71) and O_2_ (0.12 versus 0.22), indicating more efficient activation of CO and O_2_ on Pt-Ru DAPS. Combined with DFT calculations and in situ DRIFTS experiments, we confirm that the enhanced CO oxidation performance of Pt_1_Ru_1_/ND@G stems from the formation of Pt-Ru DAPS. The Pt_1_Ru_1_ bond increases the metallicity of the active sites, facilitating the simultaneous activation of CO and O_2_ while mitigating the competitive adsorption limitations typically observed in single-atom catalysts.

To understand the synergetic interaction between Pt and Ru in the Pt_1_Ru_1_/ND@G catalyst and its influence on CO oxidation reactions, density functional theory (DFT) calculations are exploited to locate the atomistic reaction pathways. Based on the experimental characterizations, we found that a model constituted by a pair of Pt_1_Ru_1_ sitting on a single layer of graphene with triple carbon vacancies agrees well with known coordination numbers, valence states, etc. (Tables S4 and S6). Because Ru is more easily oxidized than Pt, Ru presents more coordination with neighboring carbons, while Pt is slightly out of the graphene plane with less coordination. In the selected catalyst model, Ru bears a Bader charge of 0.54 and Pt bears a Bader charge of 0.05 (Table S6), which is consistent with the observed enhanced metallicity of Pt.

**Fig. 6:**
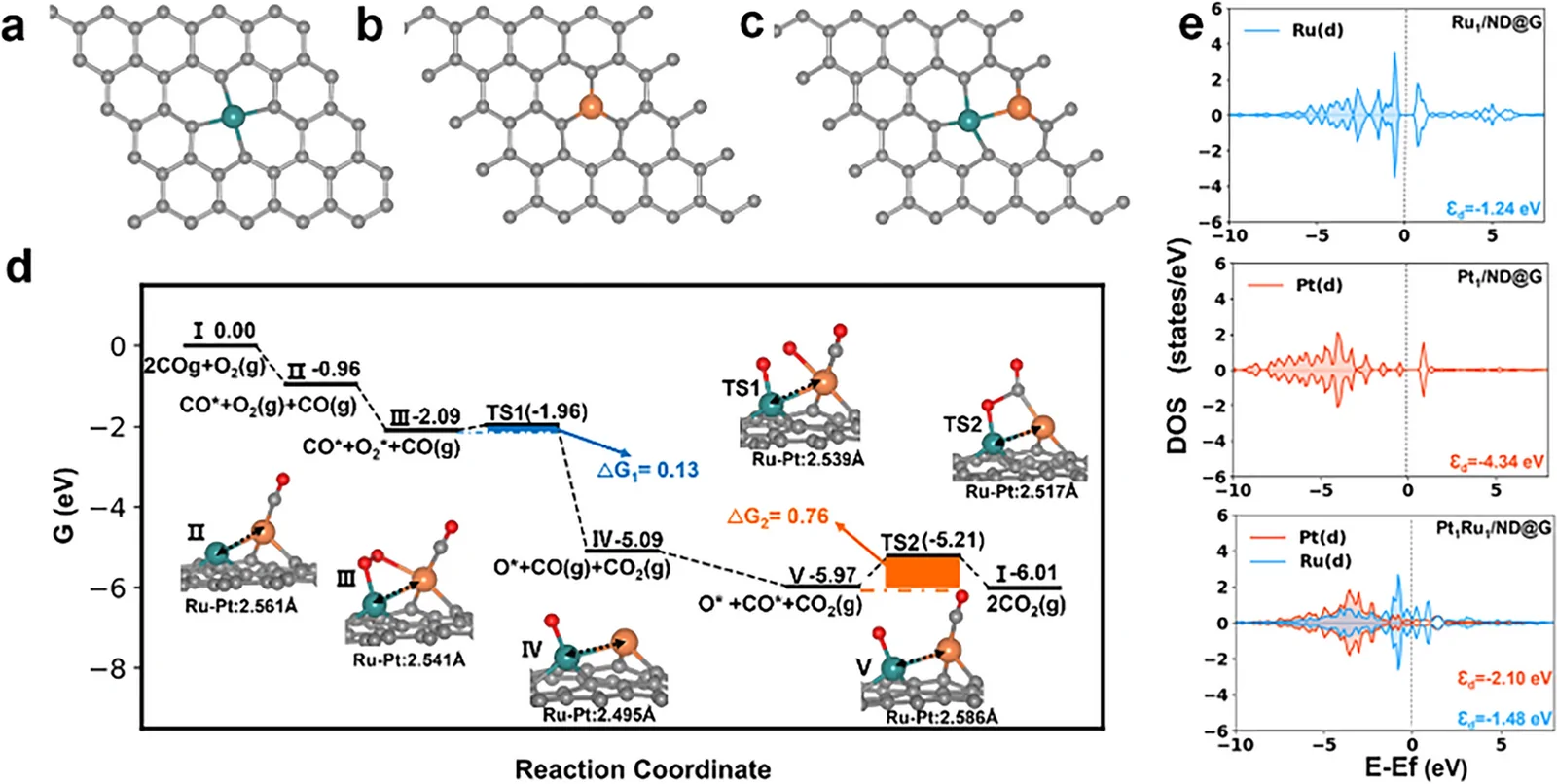
**a–c** Top views of Ru_1_/ND@G, Pt_1_/ND@G, and Pt_1_Ru_1_/ND@G. **d** Free energy profiles for CO oxidation on the Pt_1_Ru_1_/ND@G catalyst. (X-axis is the reaction coordinate, y-axis is the free energy determined at 50 °C, and the partial pressure for CO_2_, O_2,_ and CO is 1000, 2000, 1000 Pa, respectively.) Color codes: gray, orange, red, and green balls represent carbon, platinum, oxygen, and ruthenium, respectively. **e** Projected density of states for *d*-orbitals of Ru and Pt on Ru_1_/ND@G, Pt_1_/ND@G, and Pt_1_Ru_1_/ND@G

The computed free energy profiles for CO oxidation are shown in Fig. 6d CO first adsorbs on the less coordinated Pt site, and the subsequent O_2_ adsorbs on the bridge site of Pt-Ru. The computed absorption energy of O_2_ on Pt-Ru is − 1.91 eV (Table S7), higher than that on Pt_1_/ND@G ( − 0.96 eV) and Ru_1_/ND@G (-0.98 eV), demonstrating that the dual Pt-Ru site is superior in activating O_2_ while retaining its capability in CO adsorption. The CO adsorption and O_2_ adsorption are less competitive on the dual Pt-Ru site. The oxidation reactions proceed following the Langmuir–Hinshelwood mechanism. The first C–O bond formation leading to a desorbed CO_2_ is much more efficient on Pt_1_Ru_1_ catalyst with a small barrier of 0.13 eV (compared with a large barrier of 0.88 eV on Ru_1_/ND@G and 0.46 eV on Pt_1_/ND@G). The second CO oxidation, being the rate-determining step, is also efficiently catalyzed by Pt_1_Ru_1_/ND@G with a moderate barrier of 0.76 eV (Fig. 6d), which is significantly lower than those on Pt_1_/ND@G (1.23 eV, Fig. S15) and Ru_1_/ND@G (1.32 eV, Fig. S16).

Compared with the single Pt atom, the formation of the Pt_1_Ru_1_ dual-atom results in the upshift of the Pt d-band center from − 4.34 to  − 2.10 eV (Fig. 6e). This implies that the electron cloud of the Pt *d*-orbitals is closer to the Fermi level, thereby enhancing the interaction with CO. The increased CO adsorption capacity suggests that (Table S7) the Pt_1_Ru_1_ dual-atom catalyst may exhibit higher activity in CO oxidation reactions.

## Conclusions

In summary, we successfully developed a dual-atom Pt_1_Ru_1_/ND@G catalyst for efficient CO oxidation. The dual-atom catalyst exhibited superior CO oxidation activity compared to Pt_1_/ND@G and Ru_1_/ND@G, with the TOF values up to 17.6 × 10^−2^ s^−1^ at 30 °C, exceeding previously reported Pt-based catalysts under comparable reaction conditions. The exceptional catalytic activity is attributed to the formation of unique Pt_1_Ru_1_ dual-atom pair site, exhibiting enhanced metallicity of both Pt and Ru species for promoting the simultaneous adsorption and activation of reactants, while tuning the conventional competitive adsorption into a non-competitive pathway. This work demonstrates that Pt_1_–Ru_1_ dual-atom catalysts effectively circumvent the inherent limitations of single-atom catalysts, such as competitive adsorption of reactants on isolated sites and low electronic state density of single-atom sites, establishing a design principle to maximize atomic utilization and maintain metallicity for achieving superior catalytic performance.

## Supplementary Information

Below is the link to the electronic supplementary material.

Supplementary file1 (DOCX 7964 KB)
