# Supplementary material for: Atomically Dispersed Pt-Ru Dual-Atom Catalysts for Efficient Low-Temperature CO Oxidation Reaction
Source: Nanomicro Lett. 2026 Jan 5;18:172. doi: 10.1007/s40820-025-01997-6 (PMC12765774; doi:10.1007/s40820-025-01997-6)
Supplement: Supplementary file 1 — Supplementary file1 (DOCX 7964 KB) [file 40820_2025_1997_MOESM1_ESM.docx]

Supporting Information for

**Atomically Dispersed Pt-Ru Dual-Atom Catalysts for Efficient Low-Temperature CO Oxidation Reaction**

Yanan Qi^1, 2, #^, Hongqiu Chen^3, #^, Feng Hong^1,2, #^, Xiangbin Cai^4, #^, Zhehan Ying^5^, Jiangyong Diao^1,2^, Zhimin Jia^1,2^, Jiawei Chen^1,2^, Ning Wang^6^, Shengling Xiang^6^, Xiaowen Chen^1,2^, Guodong Wen^1, 2^, Bo Sun^1, 2, *^, Geng Sun^3*^, Hongyang Liu^1, 2, *^

1 School of Materials Science and Engineering, University of Science and Technology of China, Shenyang 110016, P. R. China

2 Shenyang National Laboratory for Materials Science, Institute of Metal Research, Chinese Academy of Sciences, Shenyang 110016, P. R. China

3 Chongqing Key Laboratory of Chemical Theory and Mechanism, School of Chemistry and Chemical Engineering, Chongqing University, Chongqing 401331, P. R. China

4 Division of Physics and Applied Physics, School of Physical and Mathematical Sciences, Nanyang Technological University, Singapore 637371, Singapore

5 Materials Characterization and Preparation Facility (GZ), The Hong Kong University of Science and Technology (Guangzhou), Guangzhou, P. R. China

6 Department of Physics and Center for Quantum Materials, Hong Kong University of Science and Technology, Kowloon, Hong Kong SAR 999077, P. R. China

^#^ Yanan Qi, Hongqiu Chen, Feng Hong, and Xiangbin Cai contributed equally to this work.

^*^ Corresponding authors. E-mail: [bosun@imr.ac.cn](mailto:bosun@imr.ac.cn) (Bo Sun); [sungengemail@cqu.edu.cn](mailto:sungengemail@cqu.edu.cn) (Geng Sun); [liuhy@imr.ac.cn](mailto:liuhy@imr.ac.cn) (Hongyang Liu)

**Supplementary Figures and Tables**

**
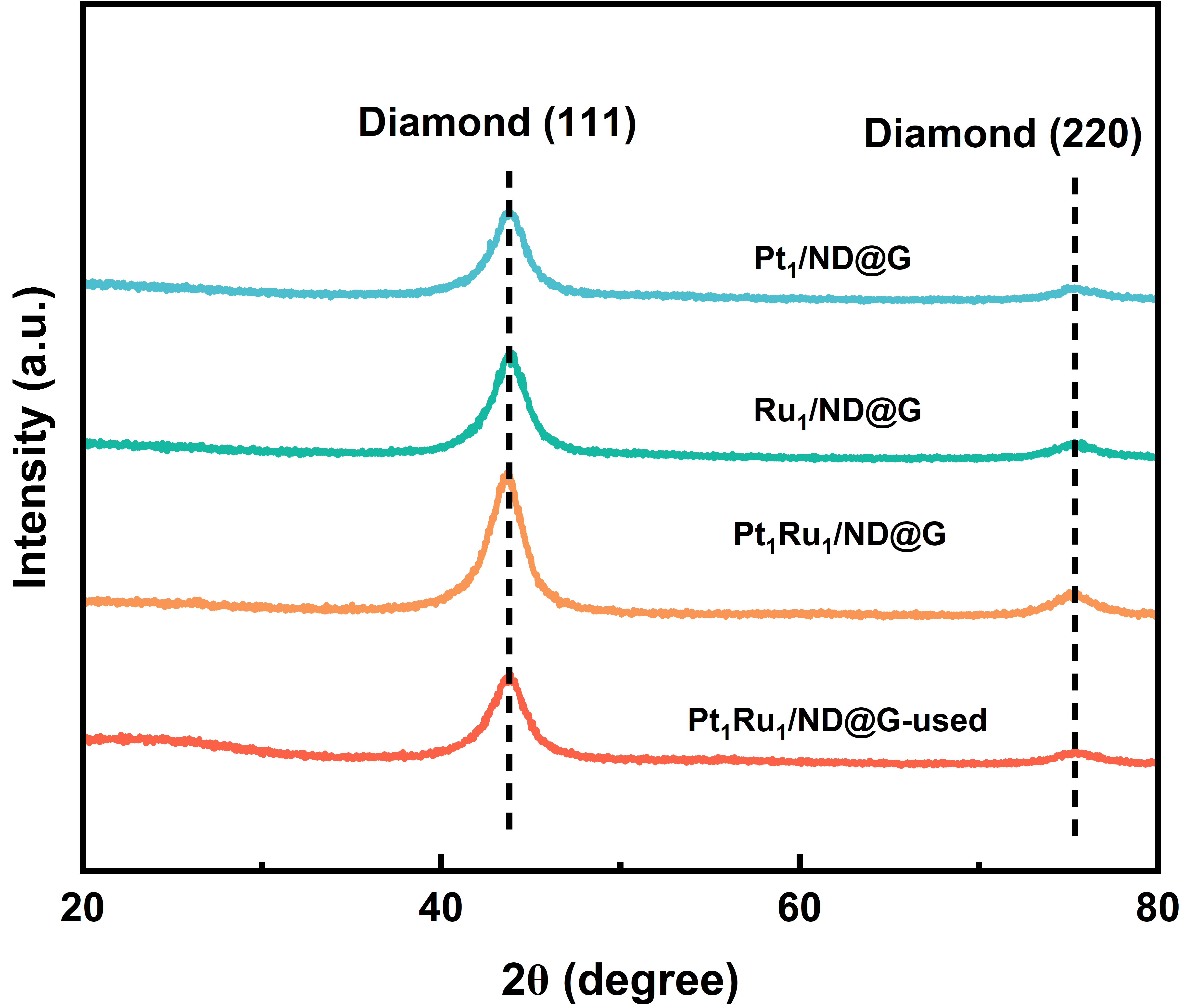
**

**Fig. S1** XRD patterns of Ru_1_/ND@G, Pt_1_/ND@G, Pt_1_Ru_1_/ND@G, and Pt_1_Ru_1_/ND@G-used


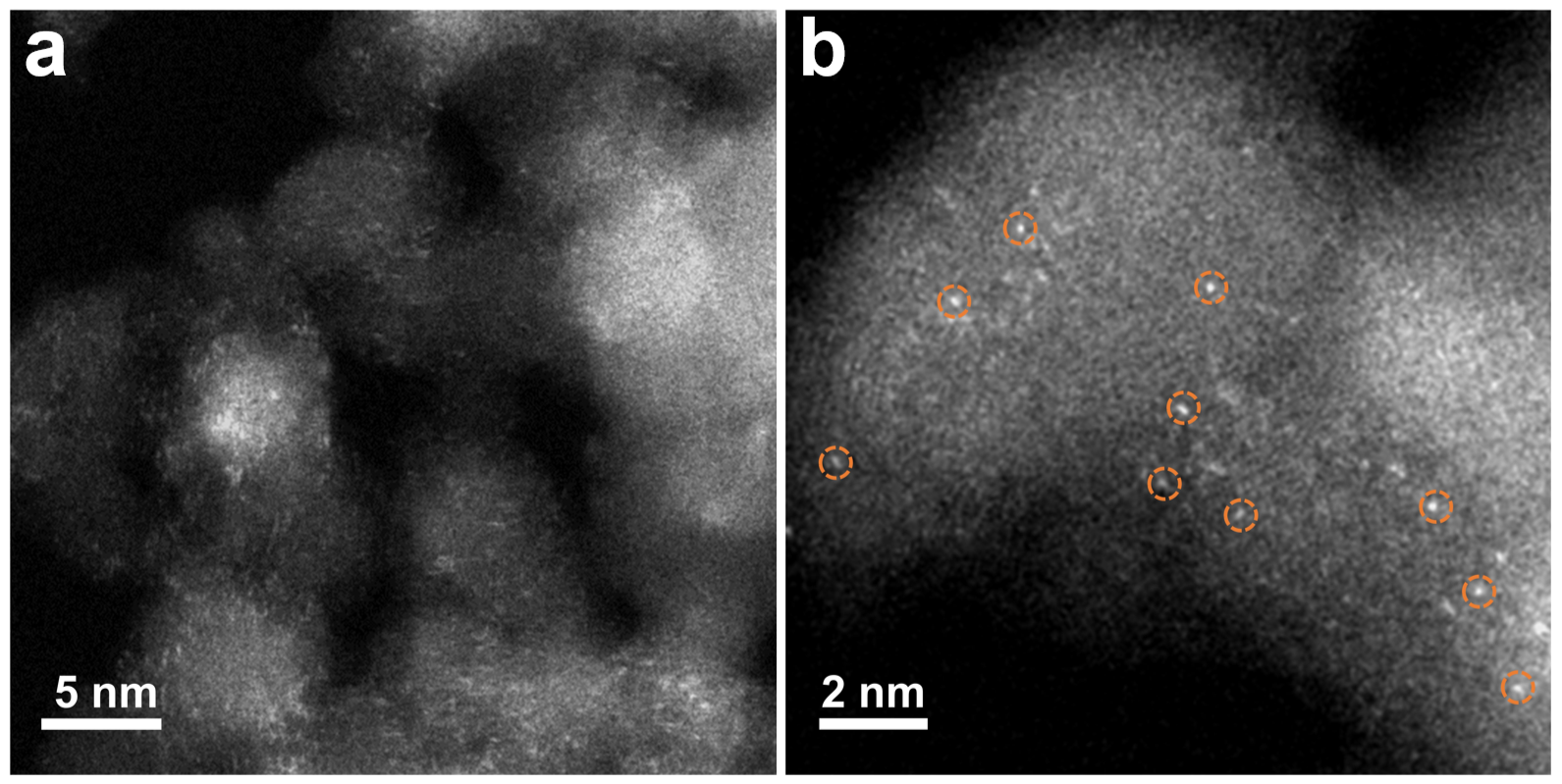


**Fig. S2** Additional HAADF-STEM images of Ru_1_/ND@G


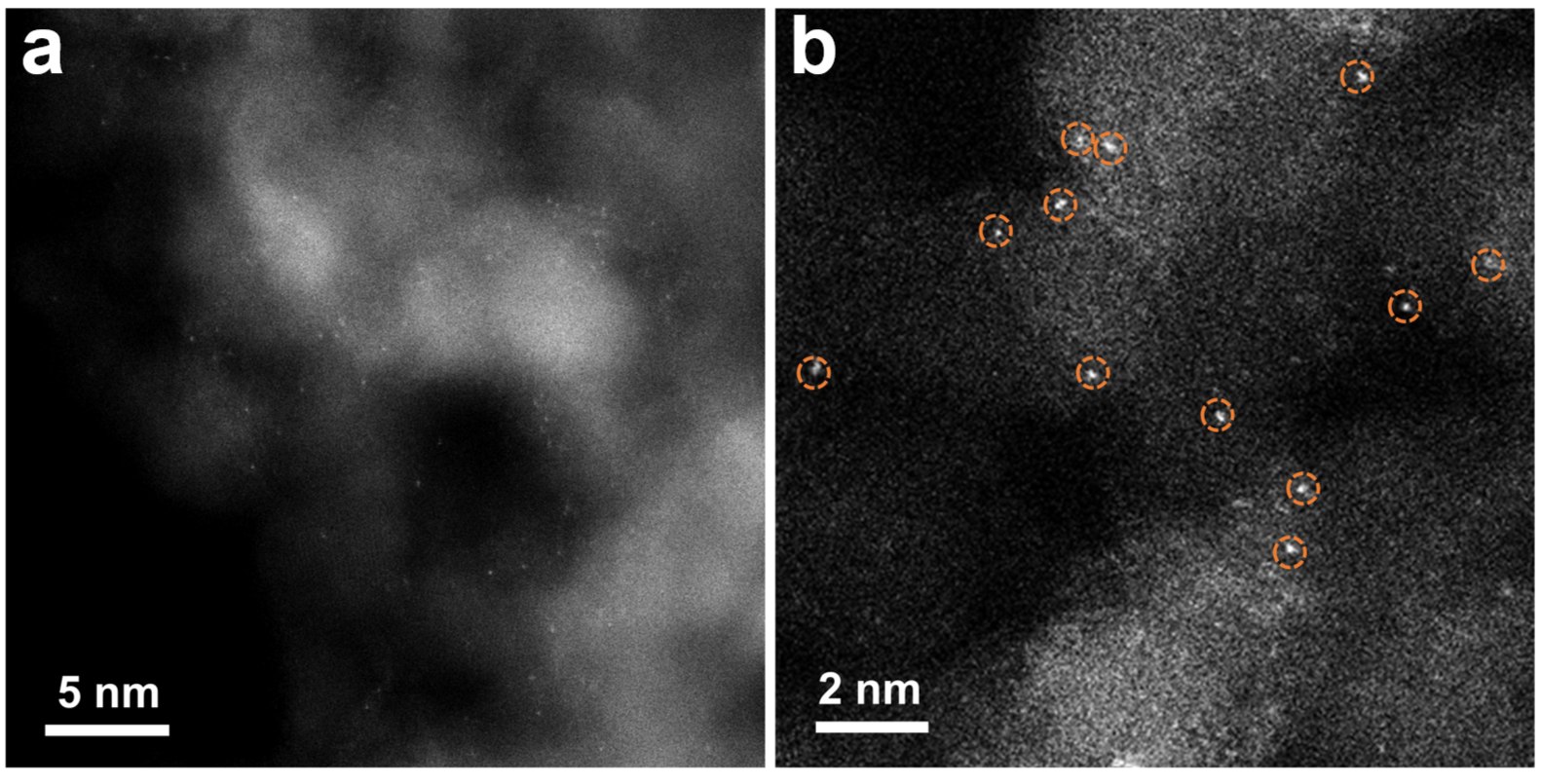


**Fig. S3** Additional HAADF-STEM images of Pt_1_/ND@G


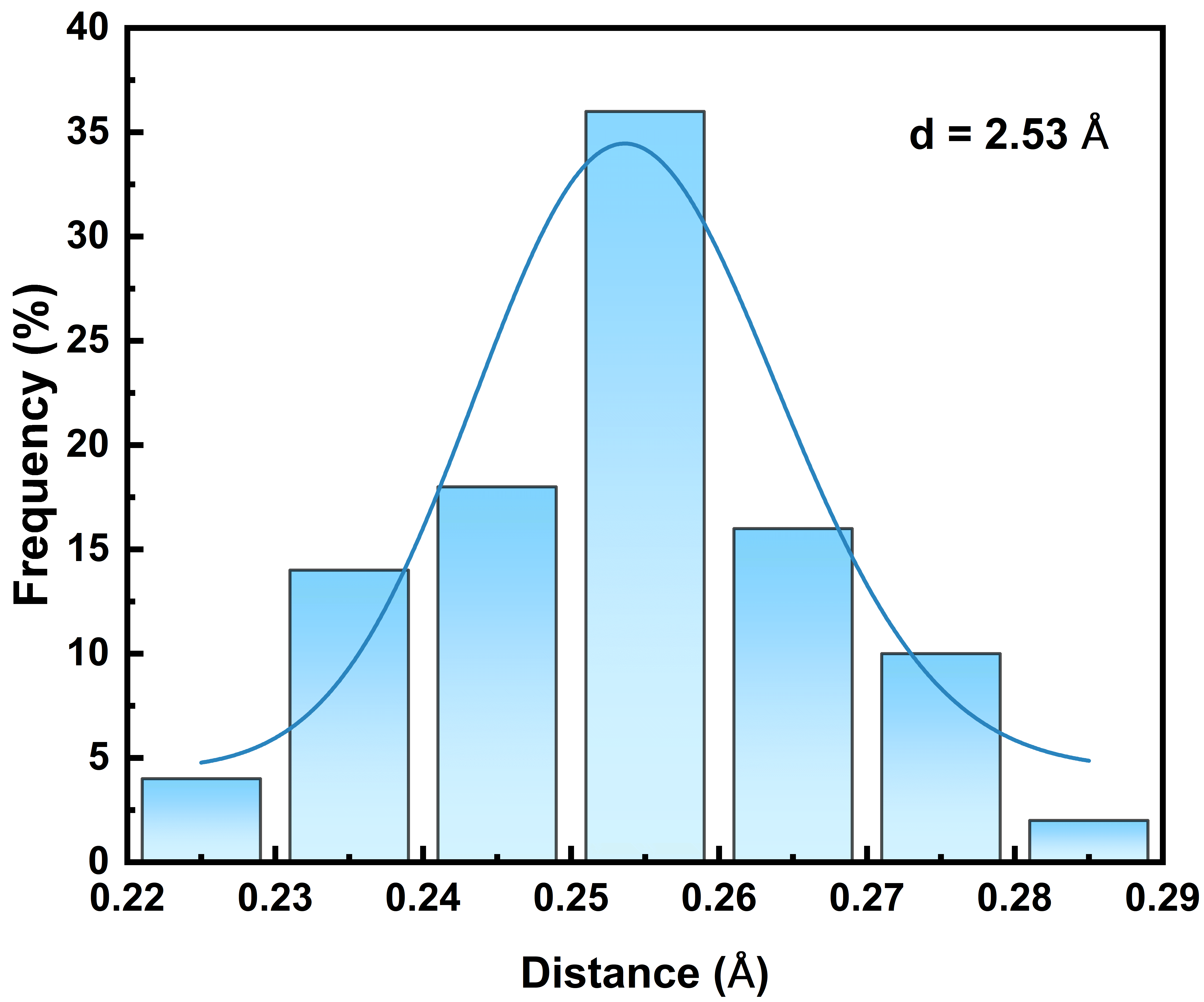


**Fig. S4** The distribution of the Pt_1_-Ru_1_ distances by counting 50 Pt-Ru atom pairs


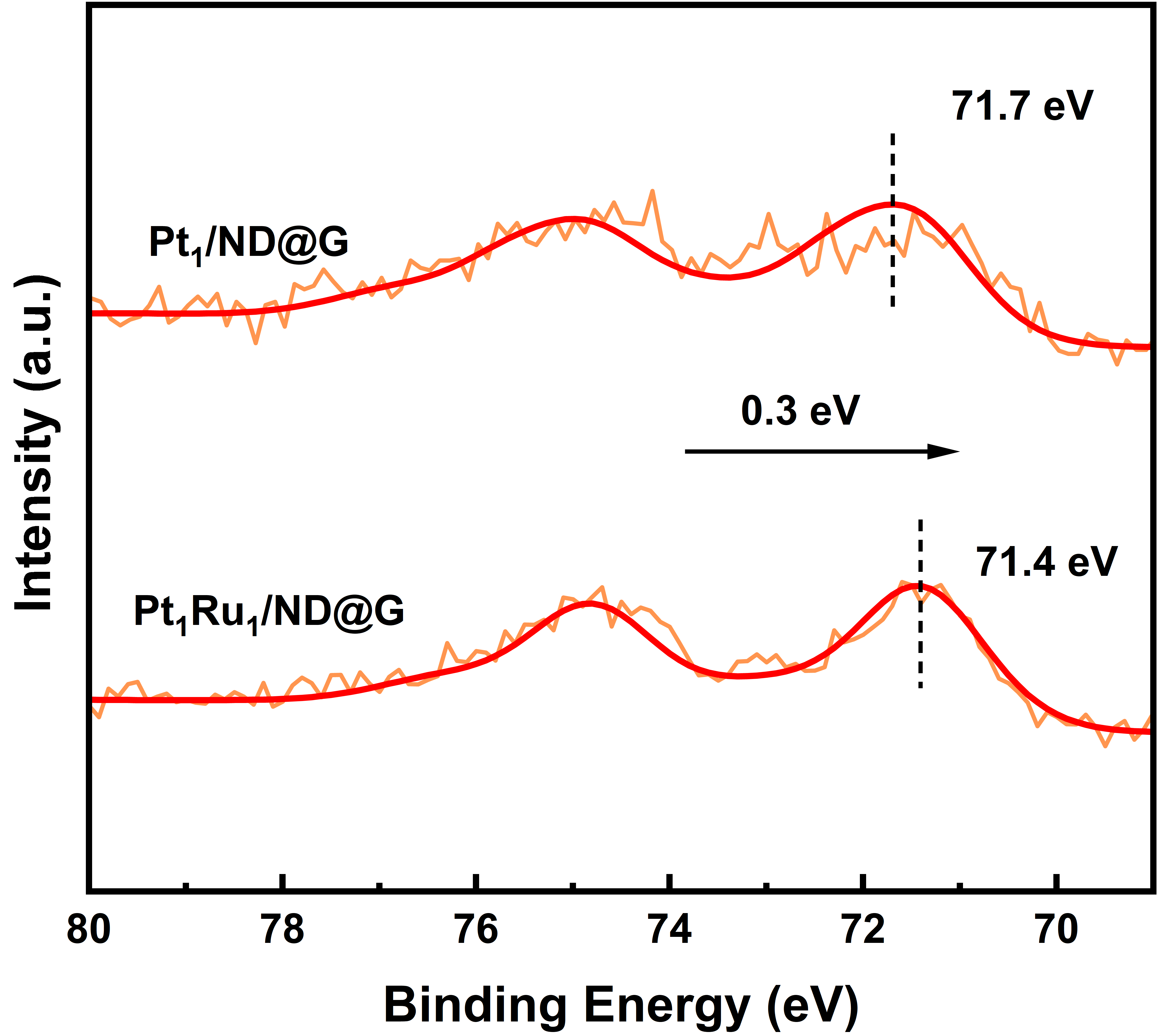


**Fig. S5** Pt 4*f* XPS spectra of Pt_1_/ND@G and Pt_1_Ru_1_/ND@G


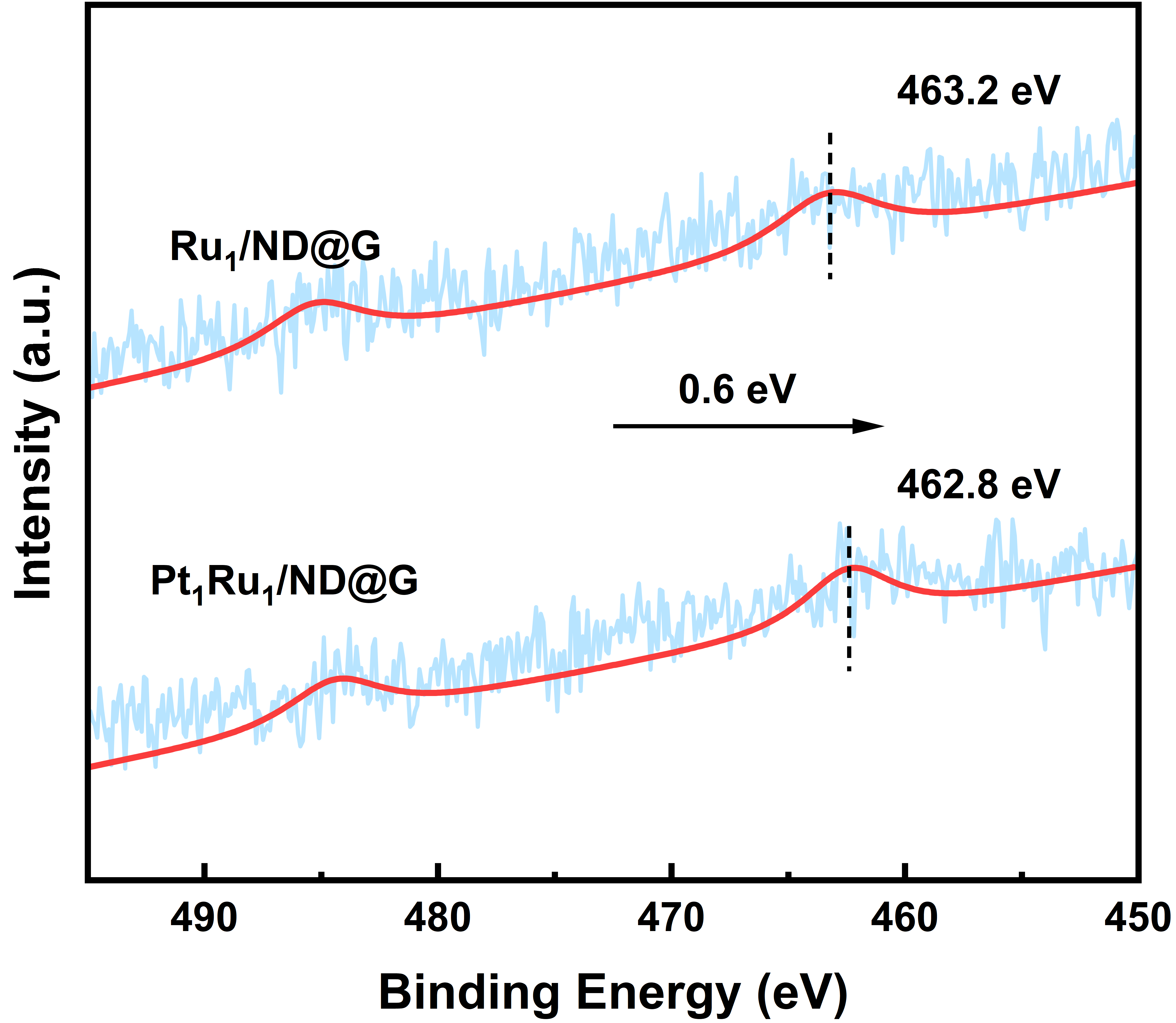


**Fig. S6** Ru 3*p* XPS spectra of Ru_1_/ND@G and Pt_1_Ru_1_/ND@G


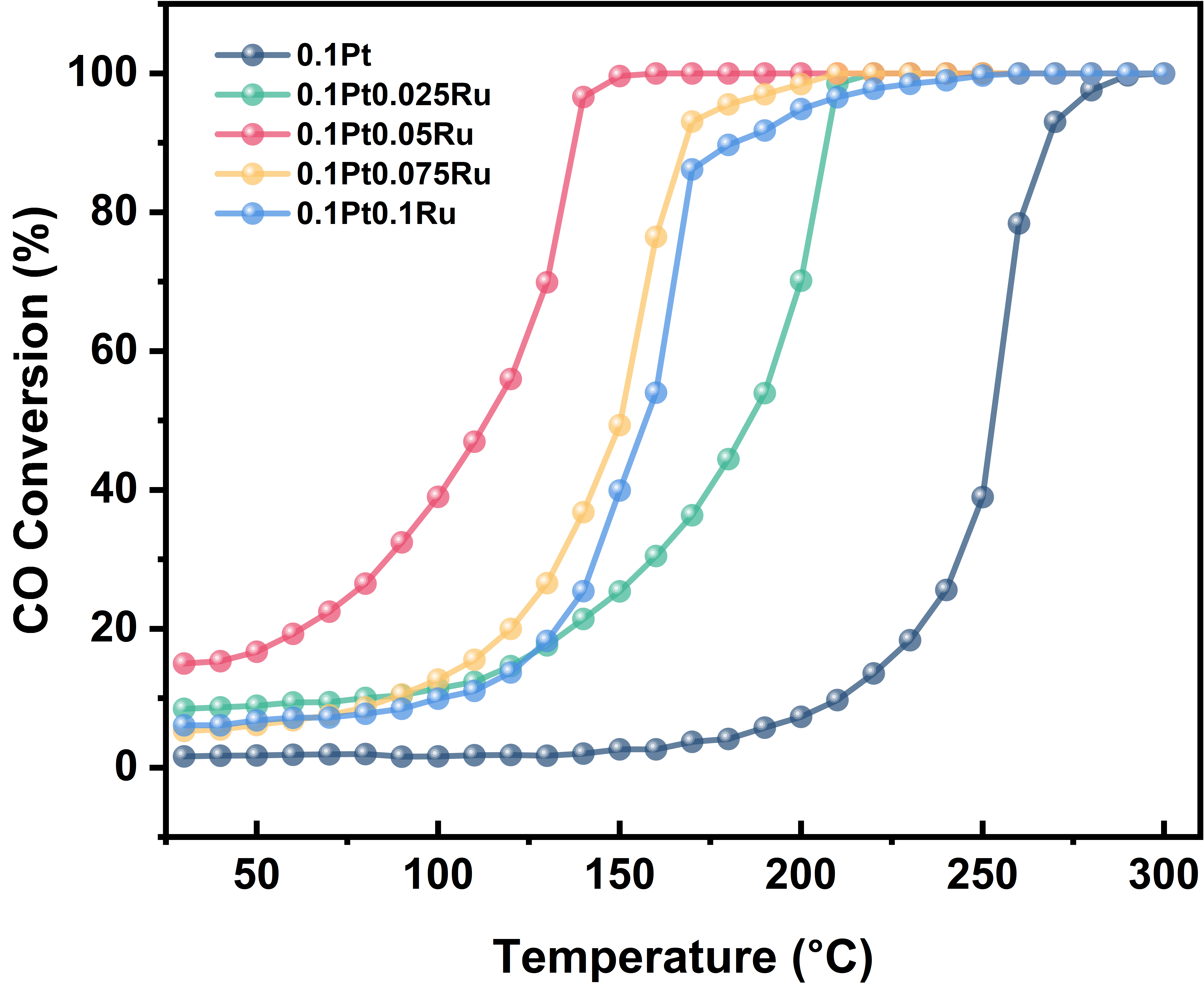


**Fig. S7** CO conversion of various PtRu/ND@G catalysts with different Ru loading. Reaction conditions: 1% CO, 1% O_2_ balanced in He, GHSV = 48,000 mL·g_cat_^-1^·h^-1^


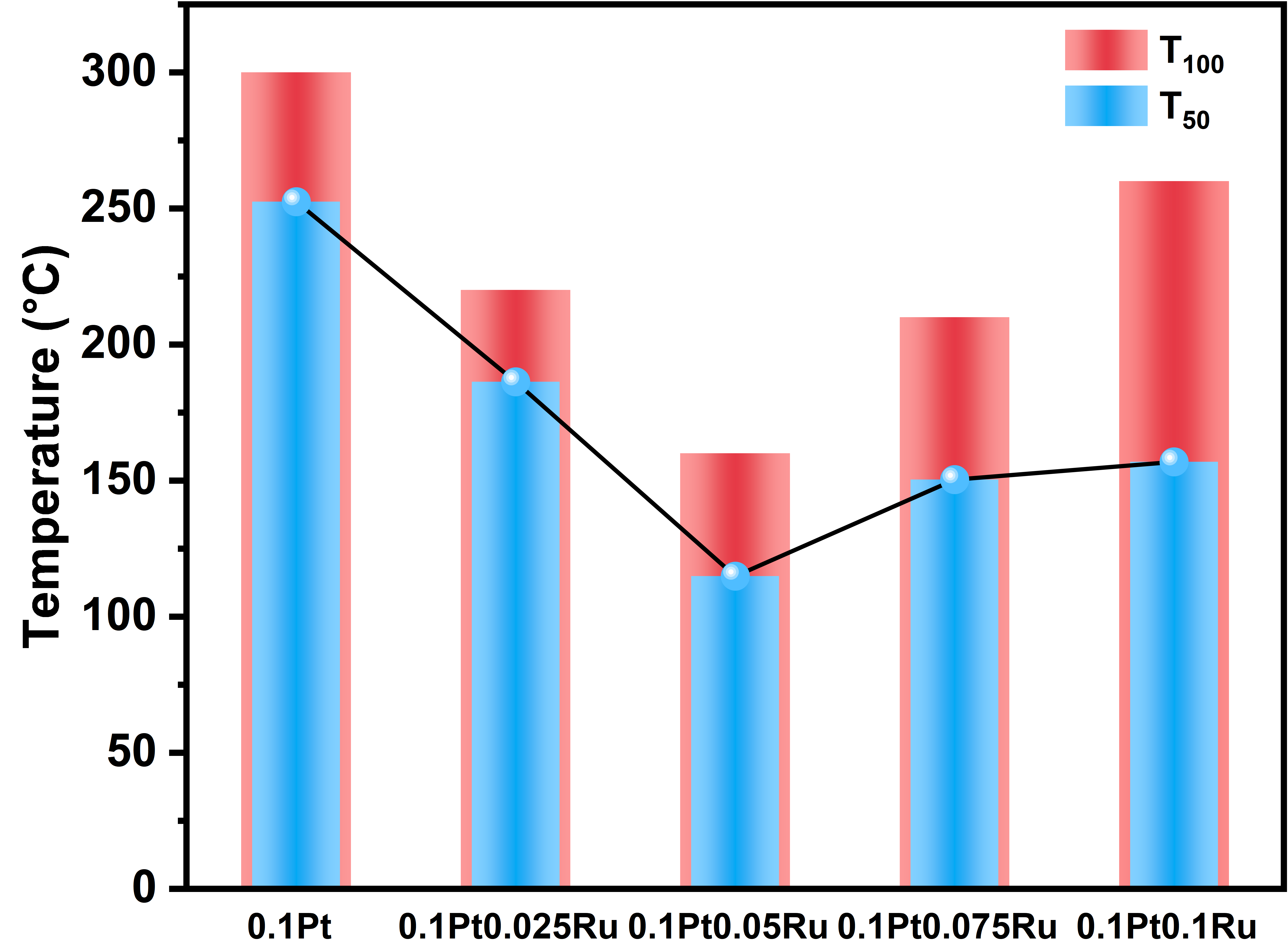


**Fig. S8** T_50_, T_100_ of various PtRu/ND@G catalysts with different Ru loading

**
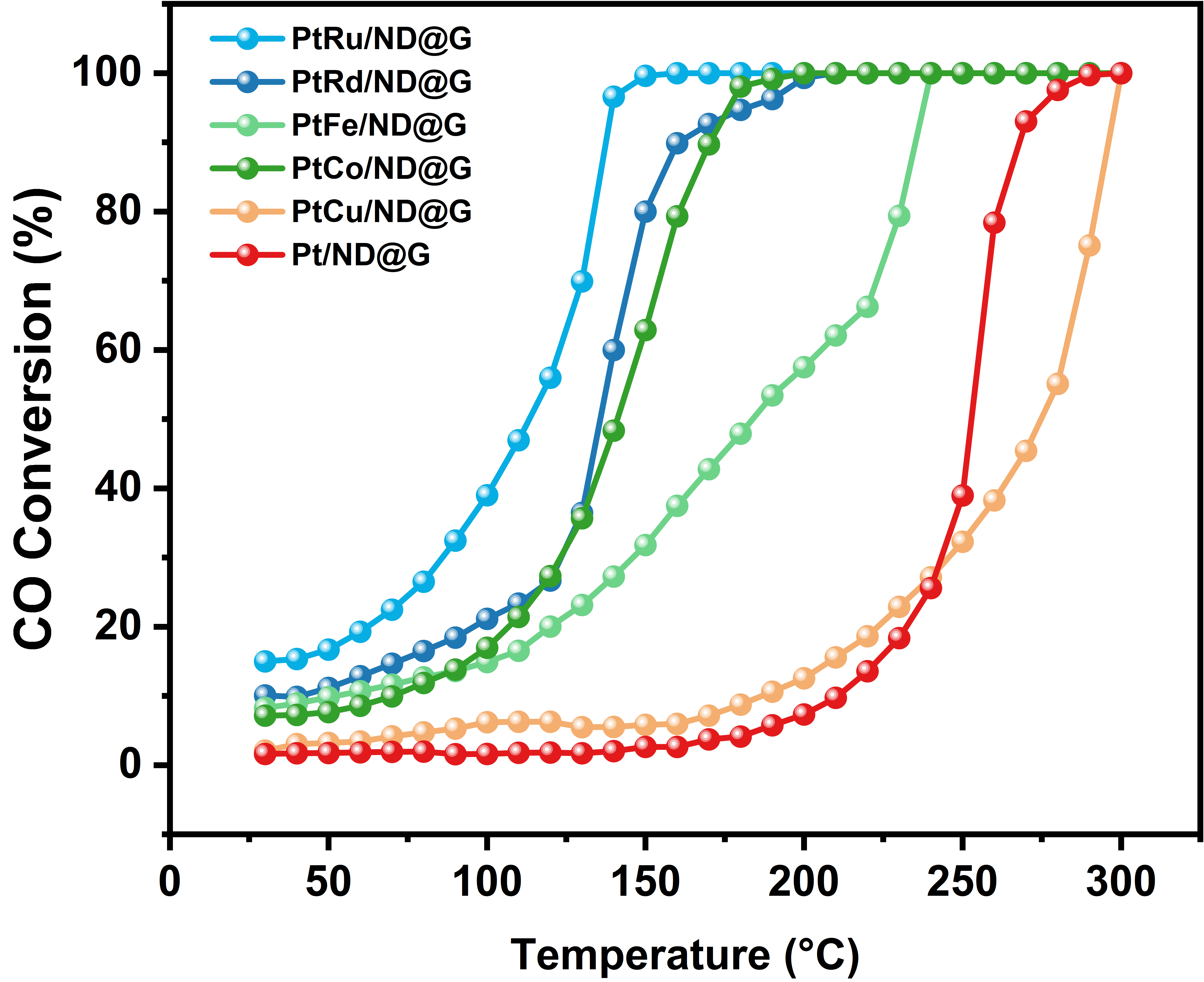
**

**Fig. S9** CO catalytic oxidation performance of different PtM/ND@G catalysts


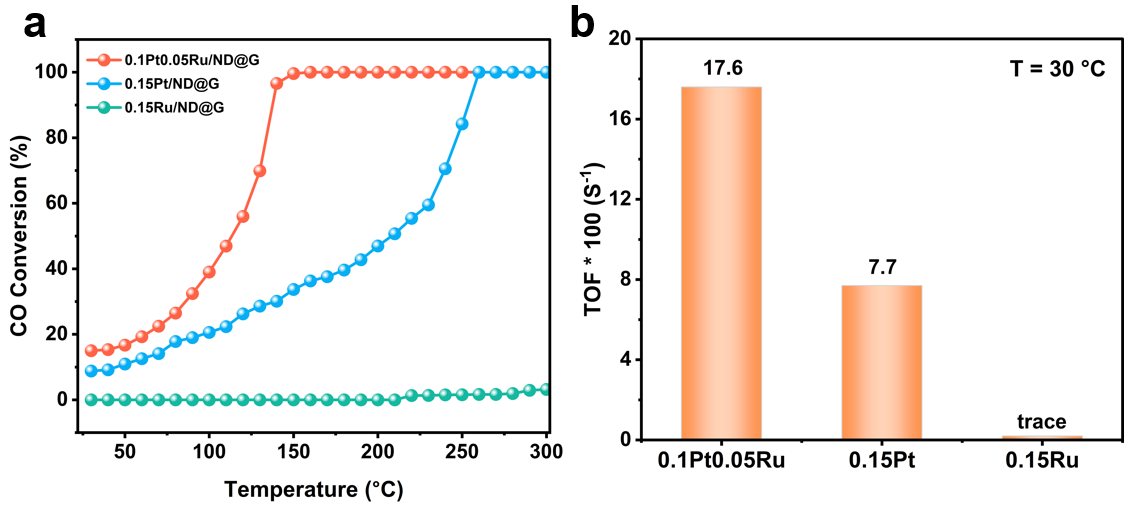


**Fig. S10** (a) Catalytic performance and (b) TOF (30°C) of Pt/ND@G, Ru/ND@G, and PtRu/ND@G with the same metal loading (GHSV = 48,000 mL·g_cat_^-1^·h^-1^)


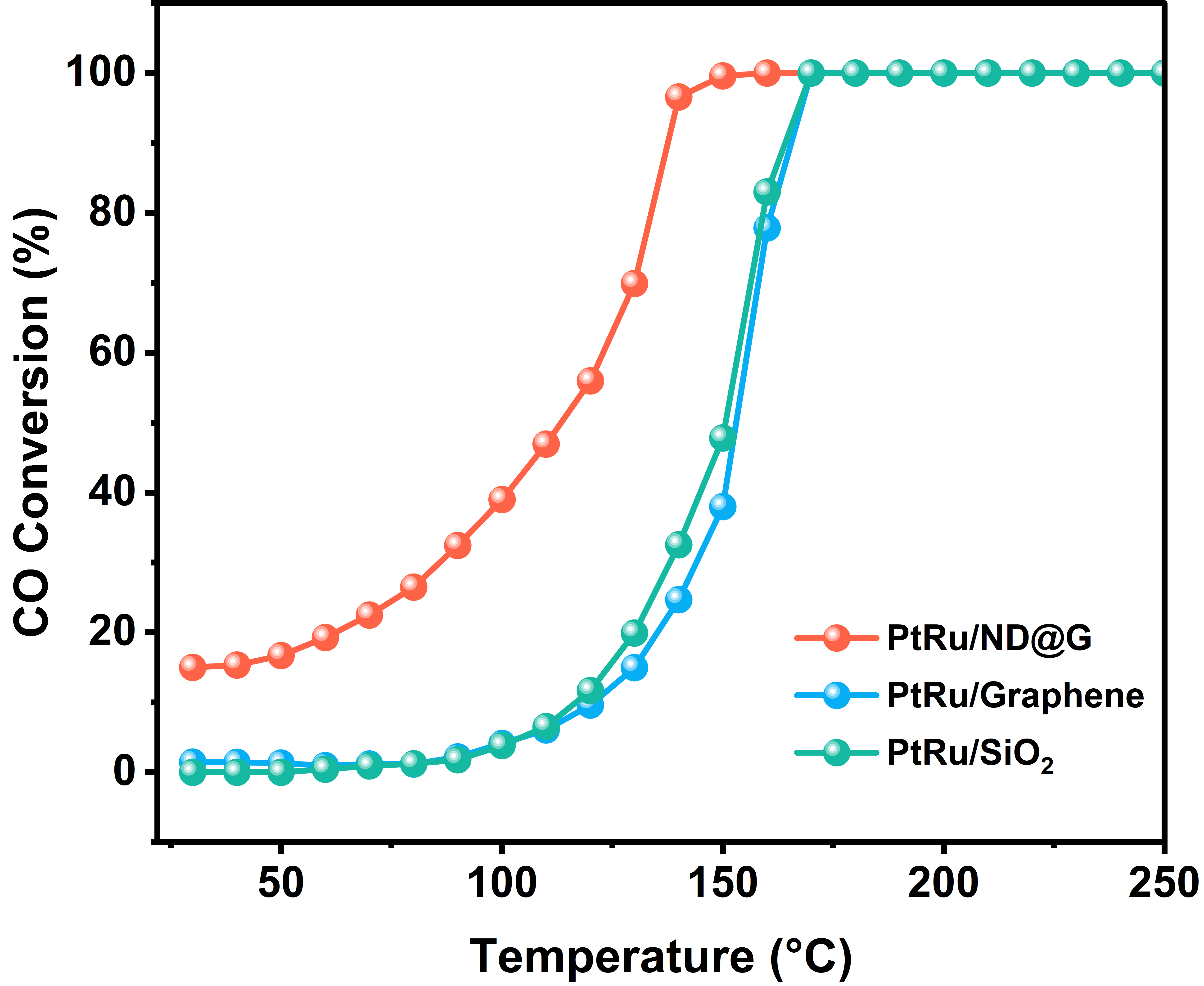


**Fig. S11** Catalytic performance of PtRu /ND@G, PtRu /Graphene, and PtRu/ SiO_2_ (GHSV = 48,000 mL·g_cat_^-1^·h^-1^)


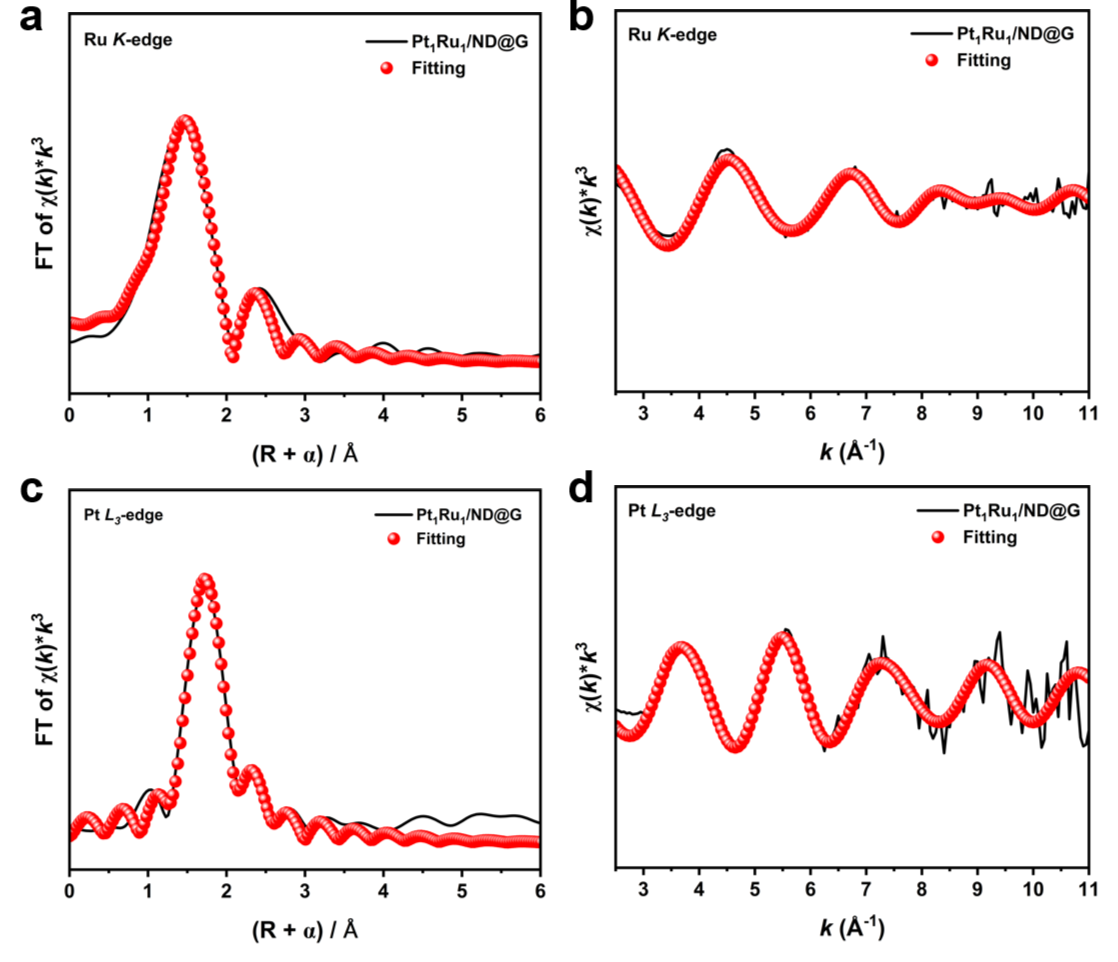


**Fig. S12** The experimental Ru (a, b) and Pt (c, d) EXAFS spectra and the fitting curves of Pt_1_Ru_1_/ND@G


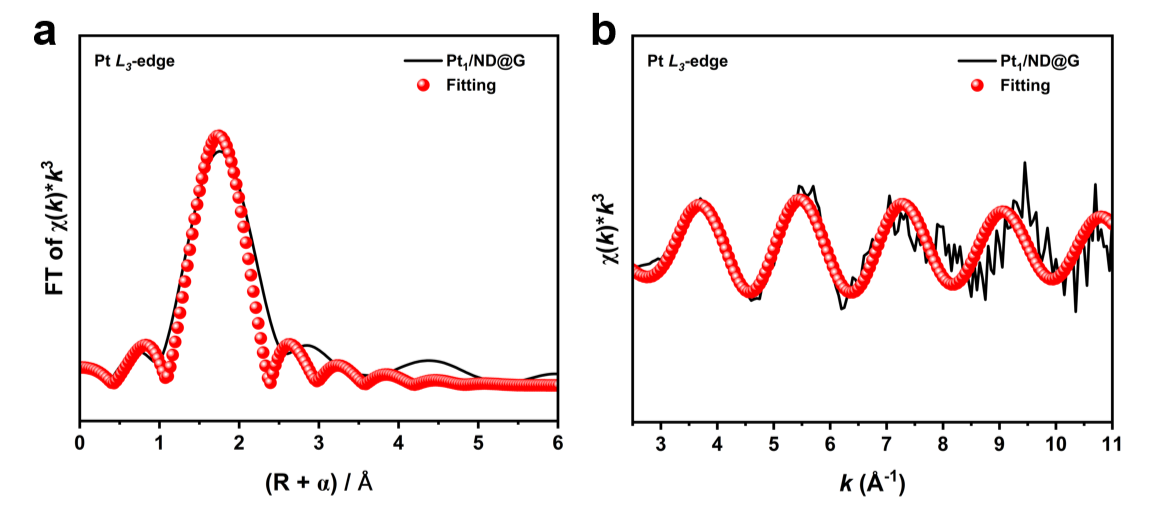


**Fig. S13** The EXAFS fitting curves of Pt_1_/ND@G at R-space


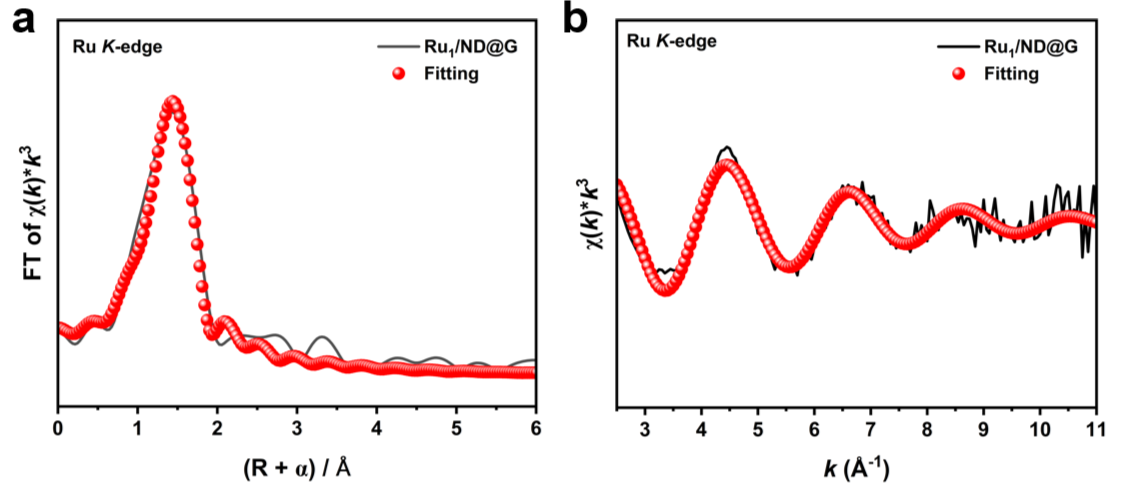


**Fig. S14** The EXAFS fitting curves of Ru_1_/ND@G at R-space


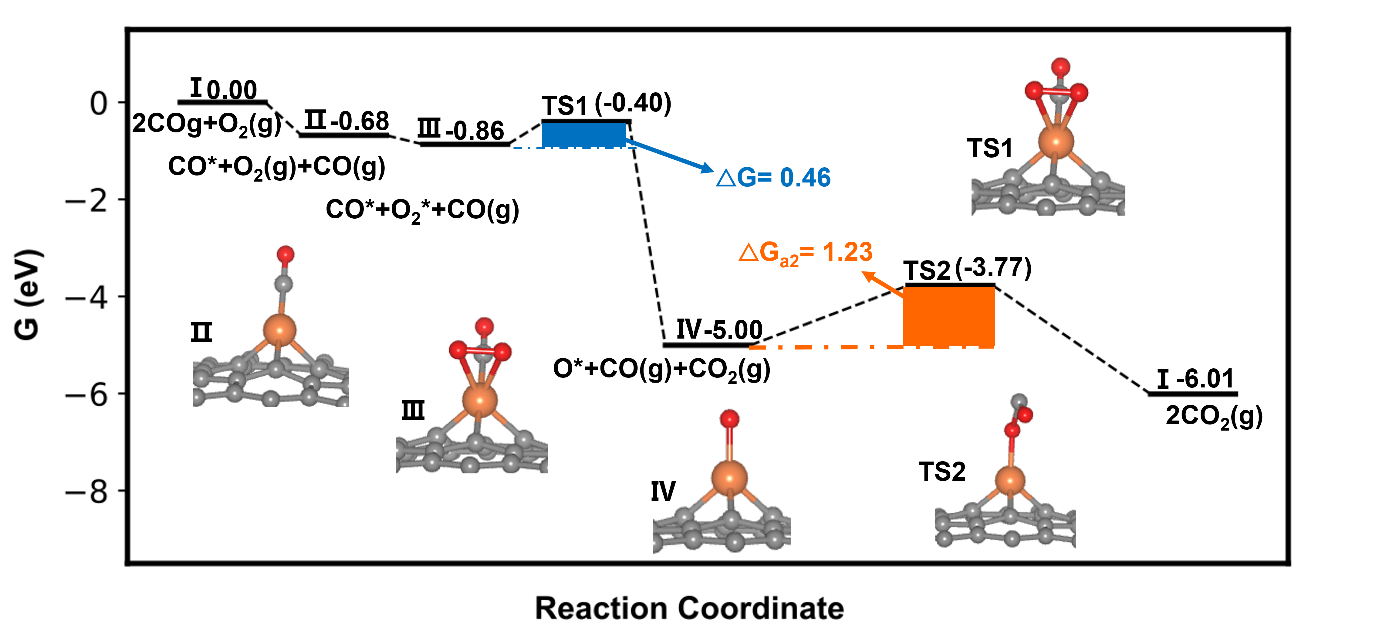


**Fig. S15** Free energy profiles for CO oxidation on the Pt_1_/ND@G catalyst (x-axis is the reaction coordinate, y-axis is the free energy determined at 50 ℃ and the partial pressure for CO_2_, O_2_ and CO is 1000, 2000, 1000 Pa, respectively. Color codes: grey, orange and red balls represent carbon, platinum and oxygen, respectively)


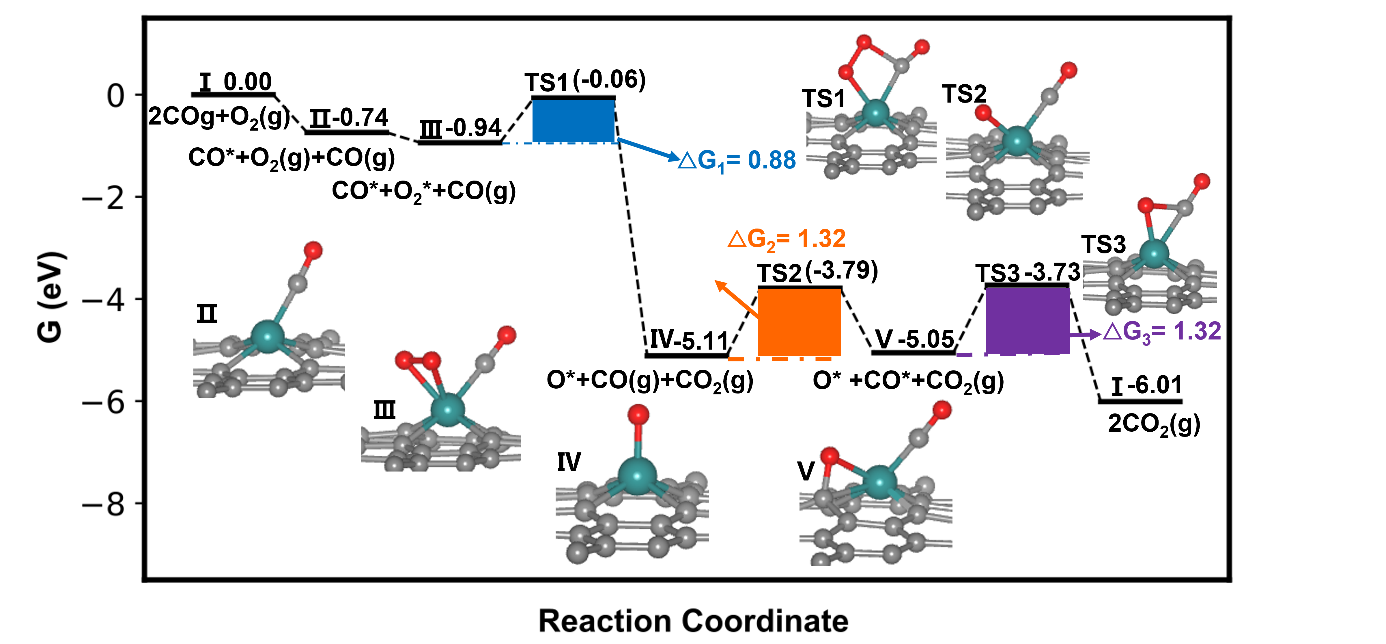


**Fig. S16** Free energy profiles for CO oxidation on the Ru_1_/ND@G catalyst (x-axis is the reaction coordinate, y-axis is the free energy determined at 50 ℃ and the partial pressure for CO_2_, O_2_ and CO is 1000, 2000, 1000 Pa, respectively. Color codes: grey, red, and green balls represent carbon, oxygen and ruthenium, respectively)


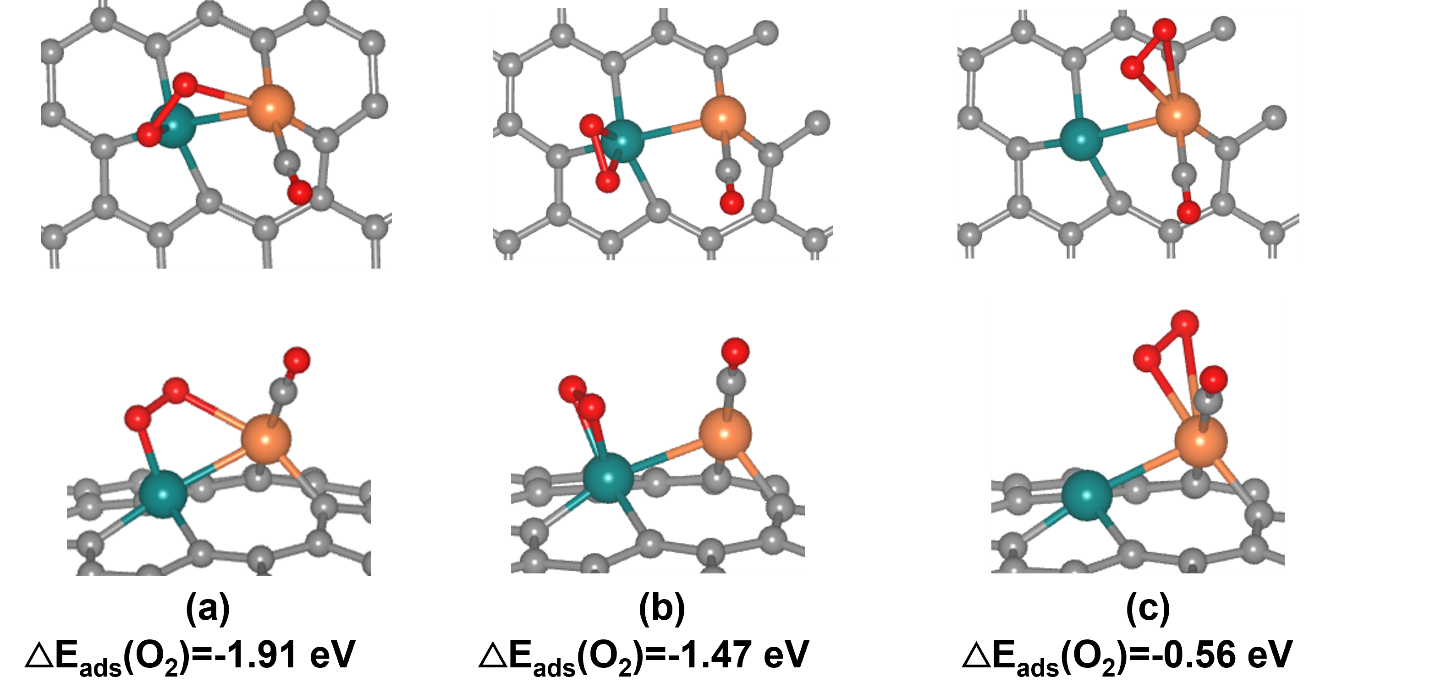


**Fig. S17** (**a**), (**b**), and (**c**) are the top and side views of the optimized structures of O_2_ adsorbed on the bridge site, Ru site, and Pt site, respectively, along with the adsorption energy of O_2_. Color codes: grey, red, orange and green balls are carbon, oxygen, platinum and ruthenium, respectively)

**
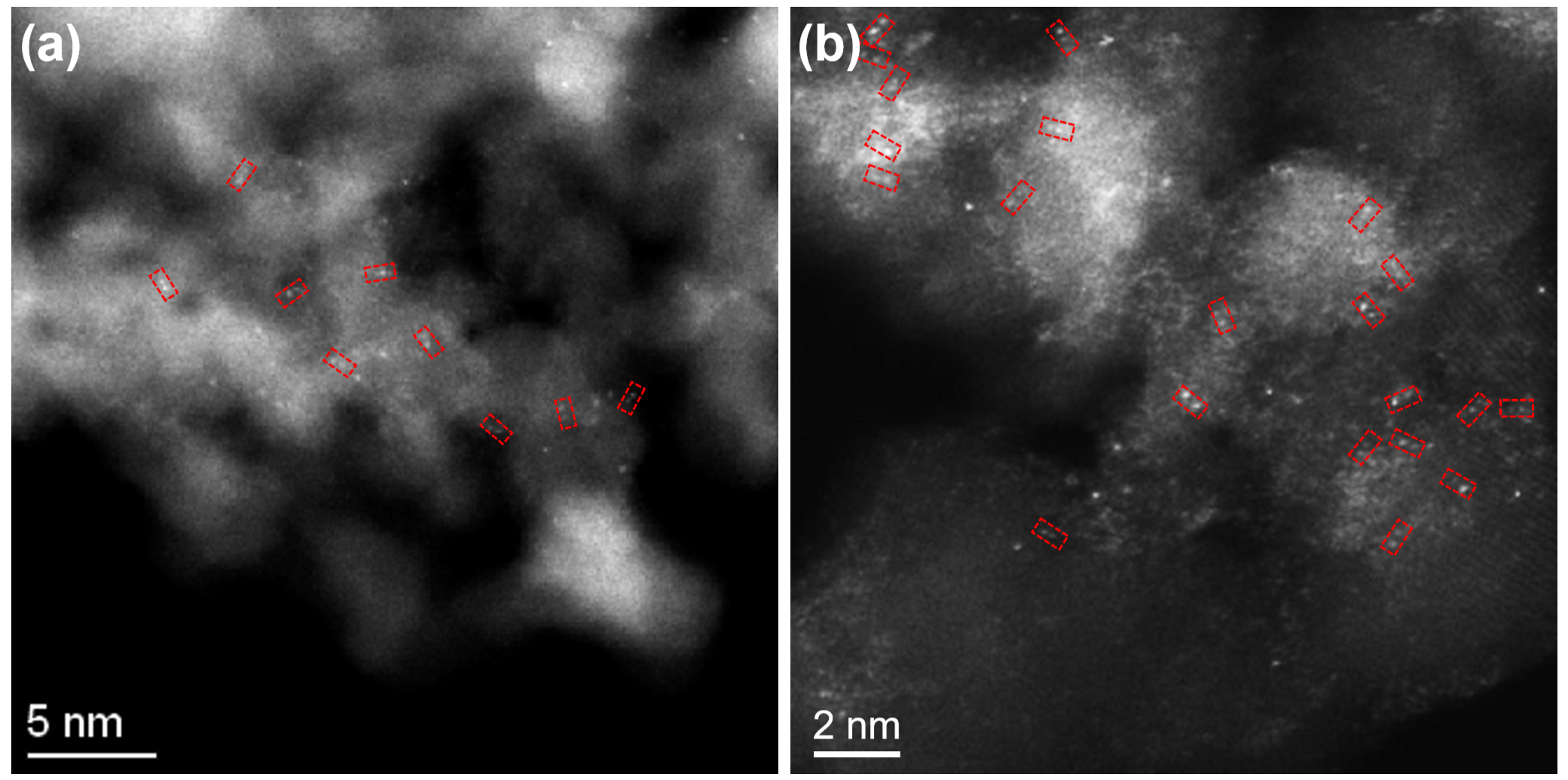
**

**Fig. S18** (**a**) and (**b**) HAADF-STEM images of Pt_1_Ru_1_/ND@G after the stability experiment

**Table S1** Comparison of the structure information and catalytic performance over the Pt_1_Ru_1_/ND@G and SACs

| **Catalyst** | **Pt loading**  **(wt%)** | **Ru loading**  **(wt%)** | **Temp.**  **(°C)** | **TOF**  ***10^2^ (s^-1^)** |
| --- | --- | --- | --- | --- |
| Ru_1_/ND@G | 0 | 0.04 | 30 | ~0 |
| Pt_1_/ND@G | 0.09 | 0 | 30 | 1.5 |
| Pt_1_Ru_1_/ND@G | 0.09 | 0.04 | 30 | 17.6 |
| Pt_1_Ru_1_/ND@G-used | 0.10 | 0.05 | 30 | \ |

**Table S2** The peak positions and shifts of platinum and ruthenium in the XPS spectra of the catalyst

| catalyst | Pt_1_/ND@G | Ru_1_/ND@G | Pt_1_ Ru_1_/ND@G |
| --- | --- | --- | --- |
| Peak Position of Pt 4f₇/₂ | 71.7 | \ | 71.4 |
| Peak Position of Ru 3p_3/2_ | \ | 463.8 | 463.2 |
| BE Shift of Pt (eV) | Ref. | \ | - 0.3 |
| BE Shift of Ru (eV) | \ | Ref. | - 0.6 |

**Table S3** The kinetic measurement parameters for catalysts in this work

| catalyst | reaction order in CO | reaction orders in O_2_ | activation energies (kJ/mol) | pre-exponential factors (h^-1^) | R^2^ values |
| --- | --- | --- | --- | --- | --- |
| Pt_1_/ND@G | + 0.71 | + 0.22 | 17.9 ± 0.6 | 221.4 | 0.99 |
| Ru_1_/ND@G | \ | \ | 23.4 ± 1.2 | 403.4 | 0.99 |
| Pt_1_Ru_1_/ND@G | + 0.53 | + 0.12 | 13.2 ± 0.4 | 492.7 | 0.99 |

**Table S4** Fit results for the EXAFS spectra of different catalysts

| **Sample** | **Path** | **R (Å)^a^** | **C.N.^b^** | **σ^2^ (Å^2^)^c^** | **ΔE_0_(eV)^d^** | **R-factor^e^** |
| --- | --- | --- | --- | --- | --- | --- |
| Ru foil | Ru-Ru | 2.68(1) | 12 | 0.004 | -3.8(17) | 0.008 |
| RuO_2_ | Ru-C/O | 1.97(1) | 6 | 0.002 | -3.2(22) | 0.018 |
| Ru_1_/NDG | Ru-C/O | 2.02(2) | 4.5 | 0.008 | -0.3(23) | 0.012 |
| Pt_1_Ru_1_/NDG | Ru-C/O | 2.02(4) | 3.9 | 0.009 | 1.3(36) | 0.018 |
|  | Ru-Pt | 2.58(4) | 0.9 | 0.003 |  |  |
| Pt foil | Pt-Pt | 2.76(1) | 12 | 0.005 | 5.2(4) | 0.003 |
| PtO_2_ | Pt-C/O | 2.01(1) | 6 | 0.003 | 9.3(12) | 0.014 |
| Pt_1_/NDG | Pt-C/O | 2.01(4) | 2.4 | 0.009 | 9.8(19) | 0.019 |
|  | Pt-Cl | 2.32(3) | 1.5 | 0.009 |  |  |
| Pt_1_Ru_1_/NDG | Pt-C/O | 2.02(2) | 1.8 | 0.004 | 9.4(26) | 0.011 |
|  | Pt-Cl | 2.28(3) | 1.2 | 0.004 |  |  |
|  | Pt-Ru | 2.52(3) | 1.1 | 0.007 |  |  |

a: R is the interatomic distance (the bond length between Pt/Ru central atoms and surrounding coordination atoms). b: CN is the coordination number. c: σ^2^ is the Debye-Waller factor (a measure of thermal and static disorder in absorber scatterer distances). d: ΔE_0_ is the edge energy shift (the difference between the zero kinetic energy value of the sample and that of the theoretical model). e: R factor is used to value the goodness of the fitting.

**Table S5** Catalytic performance over various catalysts reported in literature

| **Catalyst** | **Pt loading**  **(wt%)** | **Reaction condition** | **Temp.**  **(°C)** | **TOF×10^2^**  **(s^-1^)** | **Refs.** |
| --- | --- | --- | --- | --- | --- |
| Pt_1_Ru_1_/ND@G | 0.1 | 1% CO，1% O_2_ | 30 | 17.6 | This work |
| 0.75Pt0.2Fe/ND@G | 0.75 | 1% CO，1% O_2_ | 30 | 15.1 | [S1] |
| 0.5Pt_n_/ND@G | 0.5 | 1% CO，1% O_2_ | 30 | 3.5 | [S2] |
| Pt_1_/FeOx | 0.17 | 1% CO，1% O_2_ | 27 | 13.6 | [S3] |
| Pt-SA/A-Fe_2_O_3_ | 1.2 | 1% CO，1% O_2_ | 70 | 6.87 | [S4] |
| Pt/Sn_0.2_Ti_0.8_O_2_ | 0.5 | 1% CO，1% O_2_ | 80 | 17.2 | [S5] |
| 0.3Pt/TiO_2_ | 0.3 | 1% CO，1% O_2_ | 40 | 2.15 | [S6] |
| HO-Pt/TiO_2_ | 1 | 1% CO，1% O_2_ | 70 | 11 | [S7] |
| Pt-CC/Al_2_O_3_ | 5 | 1% CO, 1% O_2_ | 120 | 2.8 | [S8] |
| Pt-CD/Al_2_O_3_ | 2.2 | 1% CO, 1% O2 | 30 | 14.8 | [S8] |
| Pt NP/TiO_2_ | 1.0 | 0.5% CO, 5% O_2_ | 115 | 1.01 | [S9] |
| Pt/CNT-600 | 1 | 1.0 % CO, 20.0 % O_2_ | 100 | 5.55 | [S10] |
| Co_3_O_4_/Pt/Al_2_O_3_ | 3.85 | 1% CO, 10% O_2_ | 60 | 2.7 | [S11] |
| 1%wt Pt/TiO_2_(B) | 1 | 0.9% CO, 24% O_2_ | 100 | 10.9 | [S12] |
| CeO_2_-IMP-Pt | 1.2 | 1% CO, 16%O_2_ | 80 | 1.2 | [S13] |

**Table S6** The structures of optimized Pt_1_Ru_1_/ND@G, Pt_1_/ND@G, and Ru_1_/ND@G

| **catalyst** | **Optimized structures** | **Bonds** | **CN^a^** | **R(Å)** | **Bader charge analaysis** | |  |
| --- | --- | --- | --- | --- | --- | --- | --- |
|  |  |  |  |  | **Pt** | **Ru** |  |
| **Pt_1_/ND@G** | 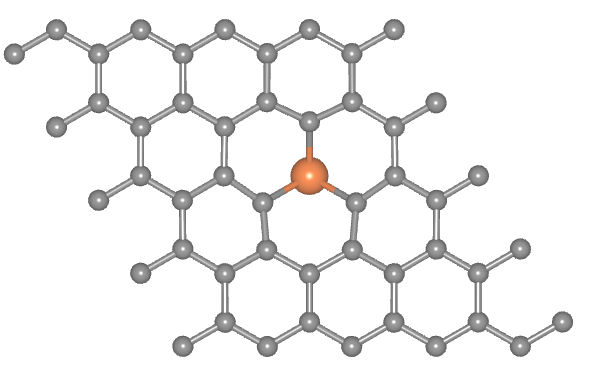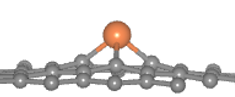 | **Pt/C** | 3 | 1.93 | **+0.22e** | \ | |
| **Ru_1_/ND@G** | 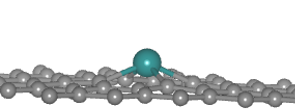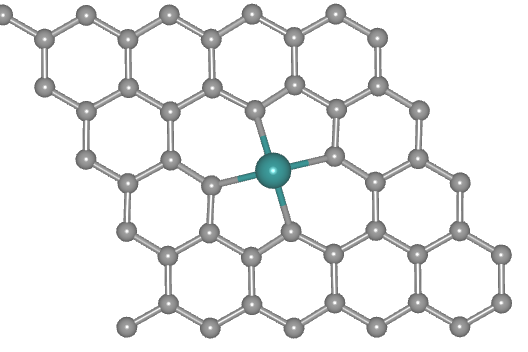 | **Ru/C** | 4 | 2.02 | \ | **+0.62e** | |
| **Pt_1_Ru_1_/ND@G** | 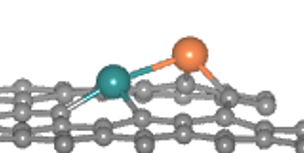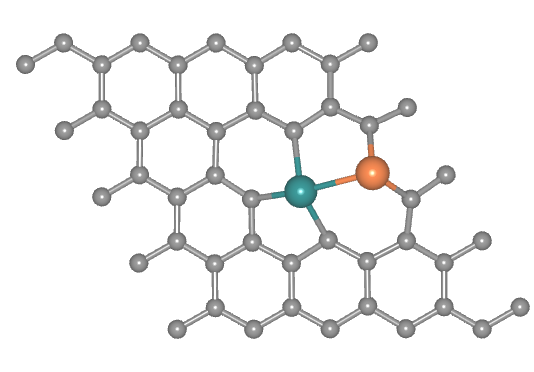 | **Ru/C** | 3 | 1.98 | **+0.05e** | **+0.54e** | |
|  |  | **Pt/C** | 2 | 1.92 |  |  |  |
|  |  | **Ru-Pt** | 1 | 2.52 |  |  |  |

^a^CN is the coordination number.

**Table S7** The adsorption energies of CO and O_2_ on Pt_1_Ru_1_/ND@G, Pt_1_/ND@G, and Ru_1_/ND@G

| **catalyst** | **Reaction** | **Eads(eV)** |
| --- | --- | --- |
| **Pt_1_Ru_1_/ND@G** | **CO(g)->CO*** | -1.74 |
|  | **CO*+O_2_(g)->CO*+O_2_*** | -1.91 |
| **Pt_1_/ND@G** | **CO(g)->CO*** | -1.46 |
|  | **CO*+O_2_(g)->CO*+O_2_*** | -0.96 |
| **Ru_1_/ND@G** | **CO(g)->CO*** | -1.52 |
|  | **CO*+O_2_(g)->CO*+O_2_*** | -0.98 |

**Supplementary References**

1. Z. Jia, X. Qin, Y. Chen, X. Cai, Z. Gao et al., Fully-exposed Pt-Fe cluster for efficient preferential oxidation of CO towards hydrogen purification, Nat. Commun. **13,** 6798 (2022). <https://doi.org/10.1038/s41467-022-34674-y>
2. Z. Jia, M. Peng, X. Cai, Y. Chen, X. Chen et al., Fully exposed platinum clusters on a nanodiamond/graphene hybrid for efficient low-temperature CO oxidation, ACS Catal. **12**, 9602–9610 (2022). <https://doi.org/10.1021/acscatal.2c02769>
3. B. Qiao, A. Wang, X. Yang, L.F. Allard, Z. Jiang et al., Single-atom catalysis of CO oxidation using Pt1/FeOx, Nat. Chem. **3**, 634–641 (2011). <https://doi.org/10.1038/nchem.1095>
4. W. Chen, Y. Ma, F. Li, L. Pan, W. Gao et al., Strong electronic interaction of amorphous Fe_2_O_3_ nanosheets with single‐atom Pt toward enhanced carbon monoxide oxidation, Adv. Funct. Mater. **29,** 1904278 (2019). <https://doi.org/10.1002/adfm.201904278>
5. J. Chen, S. Xiong, H. Liu, J. Shi, J. Mi et al., Reverse oxygen spillover triggered by CO adsorption on Sn-doped Pt/TiO2 for low-temperature CO oxidation, Nat. Commun. **14**, 3477 (2023). <https://doi.org/10.1038/s41467-023-39226-6>
6. Z. Zhou, W. Guan, X. Pan, A. Wang, T. Zhang, Synthesis of pyrazines from biomass-derived vicinal diols using ammonia over heterogeneous Pt/CeO_2_/Al_2_O_3_ catalysts, ACS Catal. **15**, 5664–5673 (2025). <https://doi.org/10.1021/acscatal.5c00372>
7. L. Zhang, X. Cheng, G. Zhang, W. Qiu, H. He et al., High active platinum clusters on titanium dioxide supports toward carbon monoxide oxidation, Appl. Catal. B Environ. **266**, 118629 (2020). <https://doi.org/10.1016/j.apcatb.2020.118629>
8. Y. Chen, Y. Feng, L. Li, J. Liu, X. Pan et al., Identification of active sites on high-performance Pt/Al_2_O_3_ catalyst for cryogenic CO oxidation, ACS Catal. **10**, 8815–8824 (2020). <https://doi.org/10.1021/acscatal.0c02253>
9. H. Hojo, M. Gondo, S. Yoshizaki, H. Einaga, Atomic and electronic structure of Pt/TiO_2_ catalysts and their relationship to catalytic activity, Nano Lett. **22,** 145–150 (2022). <https://doi.org/10.1021/acs.nanolett.1c03485>
10. W. Chen, J. Cao, J. Yang, Y. Cao, H. Zhang et al., Molecular-level insights into the electronic effects in platinum-catalyzed carbon monoxide oxidation, Nat. Commun. **12**, 6888 (2021). <https://doi.org/10.1038/s41467-021-27238-z>
11. X. Liu, Q. Zhu, Y. Lang, K. Cao, S. Chu et al., Oxide‐nanotrap‐anchored platinum nanoparticles with high activity and sintering resistance by area‐selective atomic layer deposition, Angew. Chem. Int. Ed. **56**, 1648–1652 (2017). <https://doi.org/10.1002/anie.201611559>
12. J. Liu, T. Ding, H. Zhang, G. Li, J. Ca et al., Engineering surface defects and metal–support interactions on Pt/TiO_2_(B) nanobelts to boost the catalytic oxidation of CO, Catal. Sci. Technol. **8**, 4934–4944 (2018). <https://doi.org/10.1039/C8CY01410H>
13. J. Chen, Y. Wanyan, J. Zeng, H. Fang, Z. Li et al., Surface engineering protocol to obtain an atomically dispersed Pt/CeO_2_ catalyst with high activity and stability for CO oxidation, ACS Sustain. Chem. Eng. **6**, 14054–14062 (2018). <https://doi.org/10.1021/acssuschemeng.8b02613>
